# Myco-remediation of plastic pollution: current knowledge and future prospects

**DOI:** 10.1007/s10532-023-10053-2

**Published:** 2023-09-04

**Authors:** Somanjana Khatua, Jesus Simal-Gandara, Krishnendu Acharya

**Affiliations:** 1 Department of Botany, Faculty of Science, University of Allahabad, Prayagraj, Uttar Pradesh 211002 India; 2 Nutrition and Bromatology Group, Department of Analytical Chemistry and Food Science, Faculty of Science, Universidade de Vigo, 32004 Ourense, Spain; 3 Molecular and Applied Mycology and Plant Pathology Laboratory, Department of Botany, Centre of Advanced Study, University of Calcutta, 35, Ballygunge Circular Road, Kolkata, West Bengal 700019 India

**Keywords:** Ascomycota, Bio-degradation, Biofilm formation, Fungal enzymes, Mode of action

## Abstract

To date, enumerable fungi have been reported to participate in the biodegradation of several notorious plastic materials following their isolation from soil of plastic-dumping sites, marine water, waste of mulch films, landfills, plant parts and gut of wax moth. The general mechanism begins with formation of hydrophobin and biofilm proceding to secretion of specific plastic degarding enzymes (peroxidase, hydrolase, protease and urease), penetration of three dimensional substrates and mineralization of plastic polymers into harmless products. As a result, several synthetic polymers including polyethylene, polystyrene, polypropylene, polyvinyl chloride, polyurethane and/or bio-degradable plastics have been validated to deteriorate within months through the action of a wide variety of fungal strains predominantly Ascomycota (*Alternaria*, *Aspergillus*, *Cladosporium*, *Fusarium*, *Penicillium* spp.). Understanding the potential and mode of operation of these organisms is thus of prime importance inspiring us to furnish an up to date view on all the presently known fungal strains claimed to mitigate the plastic waste problem. Future research henceforth needs to be directed towards metagenomic approach to distinguish polymer degrading microbial diversity followed by bio-augmentation to build fascinating future of waste disposal.

**Supplementary Information:**

The online version contains supplementary material available at 10.1007/s10532-023-10053-2.

## Introduction

The use of plastics is deeply embedded in our daily lives; as a result the global polymer production has increased from 1.5 to 2 million metric tonnes (MMT) in 1950 to 380 MMT by 2015 and is expected to double by 2050 (Gross and Enck 2021). Reasons behind such high popularity are low cost, water insolubility, corrosion and electricity resistance, easiness of fabrication by heating and molding, highly versatility and resilience as well as light in weight. These attributes have made plastic products better and user-friendly than other materials in a range of fields including packaging, garments, transports, consumer products, electronics, construction and industrial machinery (Urbanek et al. 2018). Recently, the demand has even been exaggerated during SARS-CoV-2 outbreak as one-time use latex gloves, surgical and face masks played important roles in protecting people from the virus (Yuan et al. 2021). The major issue with plastics is that 90% of the production consists of synthetic polymers such as polyethylene (PE), polypropylene (PP), polystyrene (PS), polyethylene terephthalate (PET), polyvinyl chloride (PVC) and polyurethane (PU) which take hundreds of years to decompose (Shams et al. 2021). For instance, plastic straw, PET water bottle, single-use diaper and PS foam are estimated to have lifetimes of 200, 450, 500 and beyond 5000 years in the milieu, respectively. Such inherent resistance to degradation coupled with excessive use has now raised a major concern regarding disposal of plastic waste (Kannan and Vimalkumar 2021).

At present, landfilling is the most widely followed technique to treat the trash that in turn causes hazards to our environment. Other practices such as incineration, recycling, pyrolysis and photo-degradation are currently showing great hope. But they are neither sufficient nor sustainable and thus has stressed on the need for more justifiable waste management protocols (Huang et al. 2022). As such, lack of proper treatment has led to the buildup of mismanaged plastic litter in all ecosystems such as soil, lakes and rivers ultimately reaching the oceans (Zeghal et al. 2021). The situation has now compelled researchers to look for alternative methods to curb plastic waste pollution. One such solution comes in the form of biodegradation that has now received great attention due to its sustainable nature and affordability (Ganesh Kumar et al. 2020). The process encompasses decomposition of plastic materials into H_2_O, CO_2_ and biomass where living organisms, microbes in particular, play a key role (Shah et al. 2008). In this context, application of fungi holds great promise as they can form biofilms on the hydrophobic plastic surface and secrete a wide range of hydrolytic enzymes that can cleave chemical bonds of the polymers. They are thus prone to attack high molecular weight (Mw) compounds and transport the small fragments through plasma membrane for further intracellular break down. Notably fungi are often regarded as better plastic degrader than bacteria as illustrated by Moyses et al. 2021; Urbanek et al. 2021; Radwan et al. 2020; Muhonja et al. 2018. Scientists across the globe are hence on the lookout for potent fungi and utilize their prowess. Till date more than 70 species, belonging predominantly to Ascomycetes, have been identified for their ability to deteriorate different synthetic as well as biodegradable plastic types (Hyde et al. 2019). Despite that, most of the reviews are focused on the fate of plastics including microplastics (MPs) (Ge et al. 2021; Issifu et al. 2020); while viewpoints and congregation of findings on polymer degradation by fungi is limited (Zimmermann 2021; Zeghal et al. 2021; Sánchez 2019). Based on the needs, the present review explicated all plastic degrading fungal strains, their enzymes and effect on different kinds of polymers. Also, a detailed understanging of fungal degradation mechanism of different kinds of plastics is brought out. Finally we confer directions of future research for succeeding widespread remedial aspect.

## Biodegradation or biotic degradation or bioremediation

**Fig. 1:**
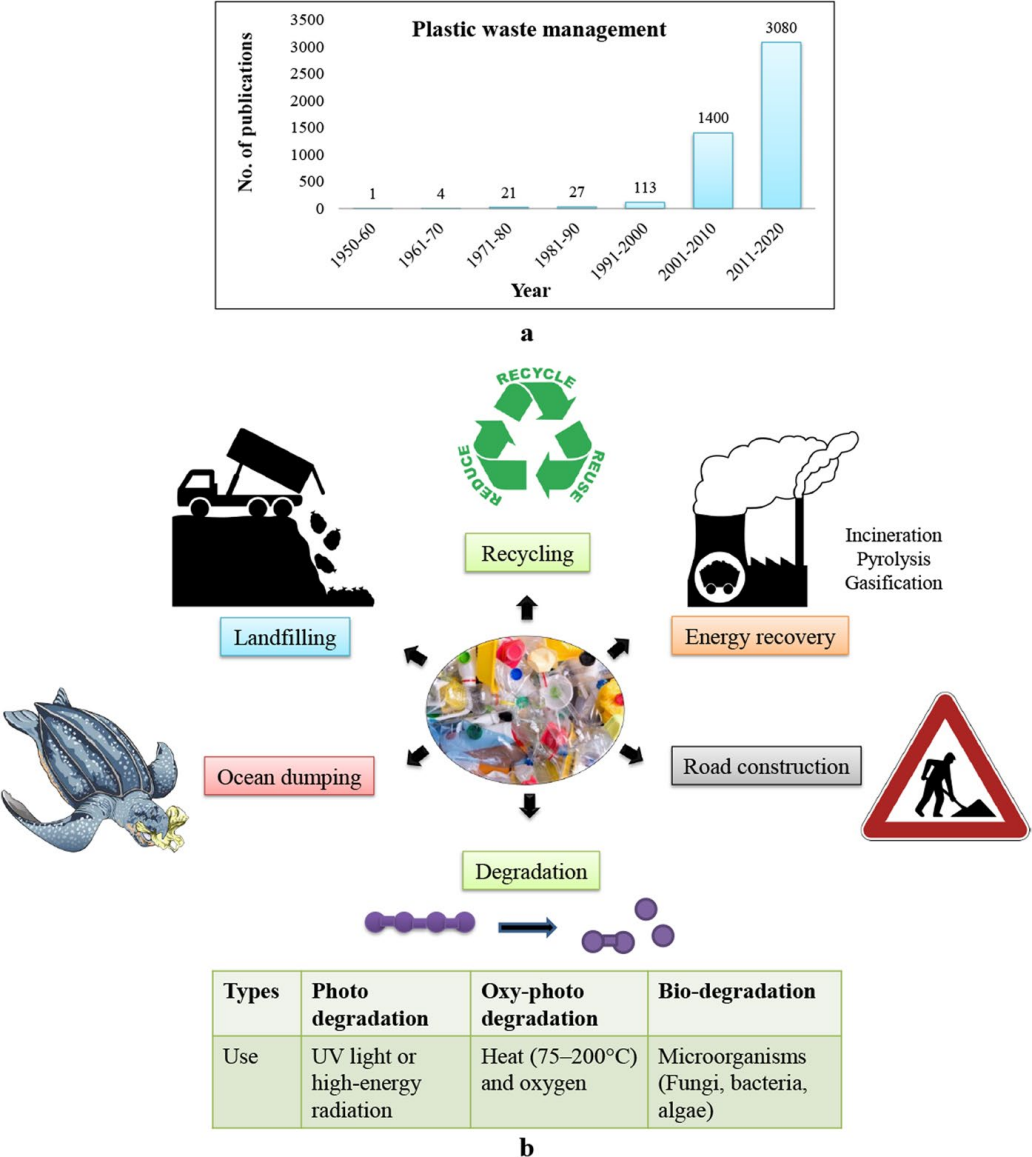
**a** Number of publications found after a literature search in PubMed with keyword “plastic waste management” (search made on 1st May, 2022). **b** Strategies to tackle plastic waste management

The continuous rising of plastic demand, human population, urbanization and economic growth led to the plastic waste production every year at an alarming rate (Miandad et al. 2019). Controlling such polymer trash is critical as unmanaged waste is not only an eyesore, but can be a breeding ground for pathogens and block sewer networks as well as water drains leading to flood. Requisite for scientific disposal of the waste was however recognized in the 1980s and since then, various attempts have been taken by numerous researchers to identify suitable methods of plastic waste management. Current techniques for addressing the problem include landfill, incineration, recycling and pyrolysis which are not only costly but also put more burdens on our environment (Huang et al. 2022). According to a study by Burt et al. (2020), the cost of mechanical removal of 25 tonnes of plastic waste was $224,537. Recently, efforts have focused on the biodegradation method being economically cheaper and more effective technique than any other traditional process of waste disposal (Zahra et al. 2010) (Fig. 1). Generally, abiotic techniques such as photo-oxidation, erosion, hydrolysis and thermal as well as mechanical stresses pave the way for biodegradation of polymers. However, the sustainable approach is largely dependent on action of microorganisms as they release hydrolytic or oxidative enzymes as well as metabolic by-products like acids (Moore-Kucera et al. 2014). Thus the process produces some economically valuable secondary products which are of great advantageous (Sanniyasi et al. 2021). Aerobic biodegradation is thus an environmentally sound method where microbial population quickly degrades heterogeneous organic matter in presence of oxygen under controlled conditions (DSouza et al. 2021).

$${\text{Organic matter}} + {\text{S}} + {\text{O}}_{{\text{2}}} \to {\text{ CO}}_{{\text{2}}} + {\text{H}}_{{\text{2}}} {\text{O}} + {\text{NO}}_{{\text{2}}} + {\text{SO}}_{{\text{2}}}$$

The course eventually causes change in optical, mechanical and electrical properties of plastics that can be assessed by measuring alteration in molar mass and tensile strength, formation of cracks, splitting, discoloration, and delamination, synthesis of new products and observing microorganism growth on the polymer substrate (Janczak et al. 2018). However, the process is governed by several factors such as polymer features and the environment where polymers are placed or disposed of (Emadian et al. 2017) (Supplementary Fig. 1). Rate of the process is also known to be affected by the type of microbes growing on the surface and their proteins. In this view, fungi have often been reported as efficient degraders of polymeric substances as the amount of proteins produced and secreted by them is significantly higher than that of bacteria (Ren et al. 2021).

## Fungi: a major recycler in the environment

**Fig. 2:**
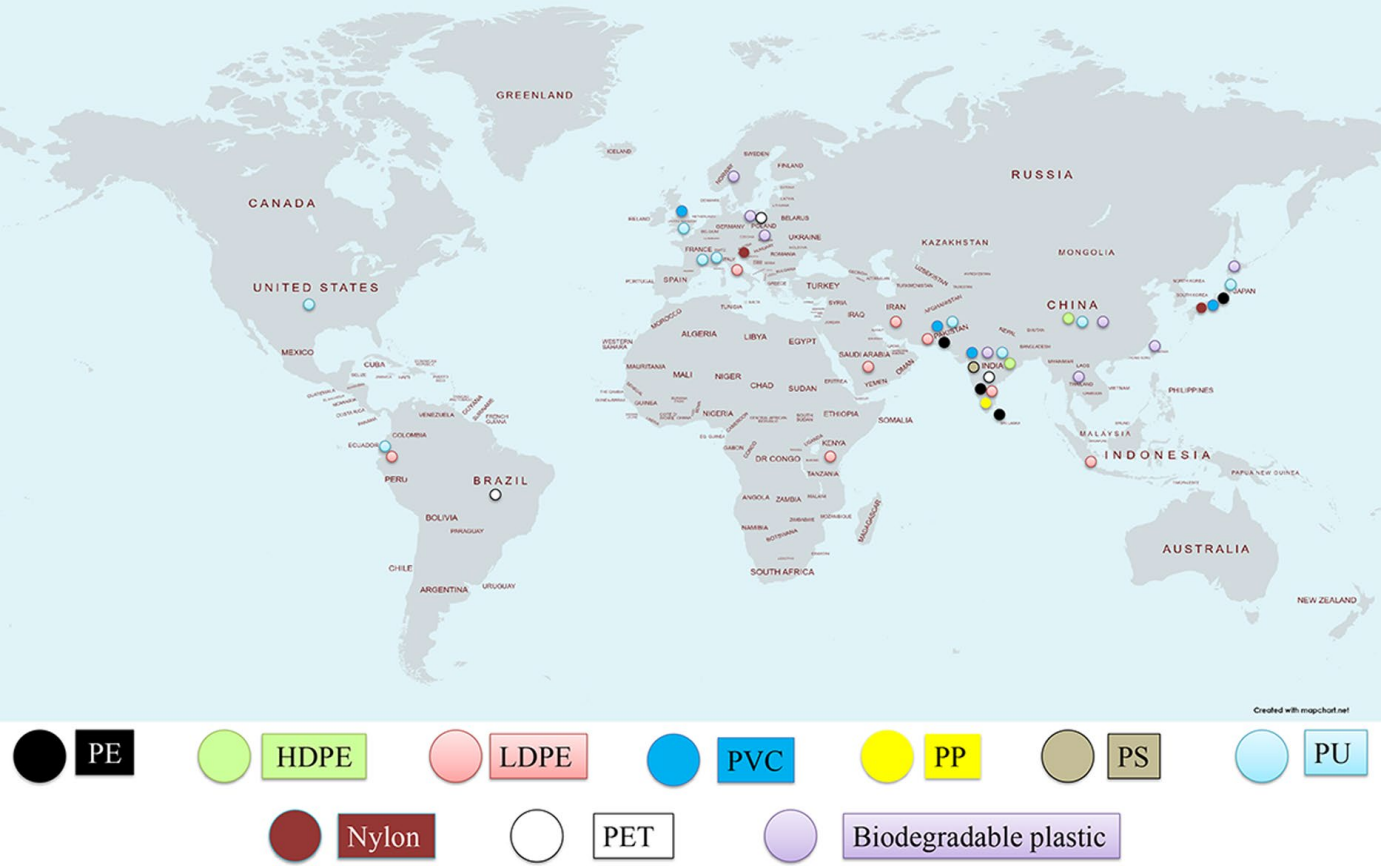
Countries reporting naturally occurring fungi potent to degrade different kinds of plastics. Most of the species occur exclusively in India, Pakistan and Japan; while, many countries remain unexplored

Fungi are a diverse group of microorganisms that inhabit various ecosystems and act as decomposers of complex constituents of plant remains, including cellulose and lignin. Indeed several species dwelling in various natural habitats and belonging to different classes has been validated to deteriorate plastics (Amobonye et al. 2021). Most of them have been reported almost exclusively from India, Pakistan and Japan (Fig. 2) (Sheth et al. 2019). Majority of the investigated taxa have been collected from marine environments due to their high abundance and ability to live on different types of floating aquatic substrates (Balabanova et al. 2018). Currently, 1901 species have been enlisted in the marine fungi website (http://www.marinefungi.org), belonging to 769 genera; although Jones (2011) suggested there may be as many as 10,000 specimens highlighting that data on fungi is still lacking. In this backdrop, Lacerda et al. (2020) designed experiments to elucidate myco-diversity associated with polymers, including micro and meso-plastics, sampled from surface water of Western South Atlantic and Antarctic Peninsula. The analysis revealed total of 64 fungal orders related with the polymers where 32 taxa were found in both the sampling locations, 21 specimens were unique to Western South Atlantic and 11 were exclusive to Antarctic Peninsula. Amongst all the specimens, genus *Aspergillus* was found to be the most prevalent taxon. Besides, *Cladosporium* and *Wallemia* have also been detected in both the places. The investigation further highlighted that some fungal groups, such as Aphelidomycota, Zoopagomycota, Mucoromycota and Blastocladiomycota had first time been reported to populate polymer in the ocaenic circumference. The observation was in accord to Wang et al. (2021) characterizing the fungal communities on three types of plastics incubated in freshwater in China. As a result, the members of Ascomycota, Basidiomycota, Blastocladiomycota and Mucoromycota were found to be the predominant communities on the tested polymers. De Tender et al. (2017) unveiled taxonomic composition of the fungal groups associated with PE sheets and dolly ropes exposed to marine environment for nearly 1 year at a harbor and an offshore place in Belgian part of North Sea. Intriguingly, no known bacterial species were detected in connection with plastic degradation; however the researchers did notice three fungal Operational Taxonomic Unit (OTU)s namely *Cladosporium cladosporioides*, *Fusarium redolens* (in higher abundance) and *Mortierella alpine*. Apart from the aquatic environment, plastic deteriorating fungi have been reported from various other sources also, like plastic dumping sites and mangrove rhizosphere soil where members belonging to the phylum of Ascomycetes and Basidiomycetes have been detected as the exceedingly potent taxa (Hock et al. 2019). Amongst them, *Penicillium* sp. and *Aspergillus* sp. have been identified as the most frequently isolated specimens possessing ability to deteriorate both biodegradable and conventional polymers (Tamoor et al. 2021) (Supplementary Table 1 and Fig. 2). Thus, reducing and eliminating plastic wastes through fungal strains represent a sustainable solution to the serious issues of environmental pollution, thanks to their unique strategies adapted for polymer disintegration and surviving ability in stressful growth conditions (Sheik et al. 2015). Detailed exploration of their potency would be extremely valuable to build unique combinations of organisms and/or enzymes to curb the problem (Sheth et al. 2019).

### Effect of fungal activity on PET

To mitigate the escalating problem of PET pollution, biodegradation merits greater attention due to its environmental friendliness and sustainability. Despite that, only few reports have been published describing ability of fungi to cause changes in the structure of PET. Malafatti-Picca et al. (2019) enumerated that two isolates of filamentous fungus namely *Microsphaeropsis arundinis* CBMAI 2109 and CBMAI 2110 could reduce PET mass by 0.5 and 0.16% respectively after 14 days of incubation. While, *Penicillium funiculosum* resulted 0.08% weight loss in PET when grown in sugar free minimal mineral medium for 84 days (Nowak et al. 2011). In a separate study, *Yarrowia lipolytica* IMUFRJ 50,682 emerged as a potent PET biodegradable organism as the yeast was found to convert PET into mono-(2-hydroxyethyl)terephthalic acid (MHET) where the PET monomers might acted as inducers in lipase production (Da Costa et al. 2020). Liu et al. (2021) engineered a PETase-expressing *Y. lipolytica* strain that hydrolyzed bis(2-hydroxyethyl) terephthalate (BHET) and PET powder into the monomers. Herrero Acero et al. (2011) showed that cutinases from *Humicola insolens*, *Pseudomonas mendocina*, *Fusarium solani*, *Thermobifida cellulosilytica* and *Thermobifida fusca* were able to hydrolyze PET. The previous studies thus suggest that enhancing catalytic activity of fungal carboxylic ester hydrolases such as cutinase and lipase could open new avenues for PET dedradation.

### Effect of fungal activity on PE

PE polymers consist of amorphous crystalline structure and are highly notorious to biodegradation due to its hydrophobicity, long carbon chains, high Mw as well as lack of functional groups resisting mineralization (Sheik et al. 2015). However, the polymers can easily be broken down via direct attack by microorganisms where the speed of degradation can be enhanced by prior UV irradiation (photo-oxidation), thermal and chemical oxidation (Ameen et al. 2015). In this context, a growing body of literature has illustrated ability of fungi to disintegrate PE of different kinds irrespective of prior treatments (Table 1). For instance Sangale et al. (2019) collected sample from plastic waste dumping sites at mangrove area. Amongst the total 109 fungal isolates, *Aspergillus terreus* and *Aspergillus sydowii* exhibited great potency in reducing plastic weight and tensile strength after 60 d of incubation. The degradation was further authenticated by scanning electron microscopy (SEM) analysis where disturbances on PE surface such as formation of cracks, scions, fissures and holes were observed confirming corrosion by the fungal strains. The similar behaviour has also been reported in case of *Zalerion maritimum*, a marine fungus, which was found to grow in minimum growth medium in presence of PE MPs (Paço et al. 2017). *Fusarium* sp. showed high potency for the degradation of PE pieces, when cultured in basal salts medium at 30 °C and 150 rpm for 2 months (Shah et al. 2009). In a separate study, Yamada-Onodera et al. (2001) suggested pretreatment of PE using *Penicillium simplicissimum* makes the deterioration process easier and faster. Similar experiments have also been performed by the same group using *P. simplicissimum* collected from local dumpsite soil in India. The organism was able to degrade UV-treated PE (38%) more efficiently than autoclaved (16%) and surface sterilized (7.7%) after 3 months of incubation. The weight loss results were in accordance to SEM observation and FT-IR analysis. Enzymes responsible for PE degradation were identified as laccase and manganese-dependent peroxidase (MnP) (Sowmya et al. 2015).

**Table 1:** Current research on degradation of plastic by fungi

Fungi	Source	Country	Plastic used	Findings	References
*Alternaria alternata* FB1	Marine	China	PE film	Morphological, chemical and physical changes; laccase and peroxidase synthesis	Gao et al. (2022)
*Aspergillus terreus, Aspergillus sydowii*	Rhizosphere soil of *Avicennia marina*	India	PE	Weight loss, decrease in tensile strength of PE, morphological and chemical changes after 60 d of growth	Sangale et al. (2019)
*Aspergillus* sp., *Fusarium* sp., *Penicillium* sp.	Dumping place	Sri lanka	PE	Weight loss, structural and morphological changes after 90 d of incubation	De Silva et al. (2019)
*Aspergillus* sp., *Penicillium* sp.	Soil	Pakistan	PE	Formed clear zone in PE media	Ahsan et al. (2016)
*Aspergillus* sp. ST-01	Soil	NM	PCL, PHB films	Fragmentation of samples within 5 d of incubation	Sanchez et al. (2000)
*Aspergillus* sp. XH0501-a	UV mutant	China	PBS	Formation of clear zone in media at 30 °C, synthesis of extracellular PBS-degrading enzyme	Li et al. (2011)
*Fusarium* sp.	Sewage sludge	Pakistan	PE	Adherence and extensive growth of fungus on PE surface, morphological changes, CO_2_ evolution after 2 months of incubation	Shah et al. (2009)
*Penicillium simplicissimum* YK	Soil and leaves	Japan	UV pretreated PE	Reduction in Mw of PE	Yamada-Onodera et al. (2001)
*Penicillium* sp., *Rhizopus arrhizus*	Soil	India	Thermally treated and UV irradiated PE	Increased biomass, weight loss after 6 months of incubation	Mahalakshmi and Andrew (2012)
*Trichoderma harzianum*	Soil from dumpsites	India	Autoclaved, UV-treated, and surface-sterilized PE	Weight loss, morphological and structural changes after 3 months of incubation; production of laccase and MnP	Sowmya et al. (2014)
*Zalerion maritimum*	Culture	Portugal	PE MPs	Reduction in size and mass of MPs after 28 d of incubation	Paço et al. (2017)
*Aspergillus flavus*	Guts of *Galleria mellonella*	China	HDPE MPs	Mw reduction and chemical changes after 28 d incubation; gene expression of laccase-like multicopper oxidases genes	Zhang et al. (2020)
*Aspergillus flavus* VRKPT2, *Aspergillus tubingensis* VRKPT1	Soil from plastic waste dumped marine coastal habitats	India	HDPE	Weight loss, structural and morphological changes, biofilm formation after 30 d incubation	Devi et al. (2015)
*Aspergillus niger* (ITCC no. 6052)	Soil from plastic waste disposal site	India	Thermally oxidized HDPE	Weight loss, reduction of tensile strength, morphological changes, biofilm formation	Mathur et al. (2011)
*Aspergillus flavus* MCP_5_, *Aspergillus flavus* MMP_10_	Dumpsite soil	Nigeria	LDPE	Weight loss, chemical changes	Kunlere et al. (2019)
*Aspergillus niger, A. terreus*	Mangrove soil	Ecuador	LDPE film	Fungal colonization, weight loss after 77 d of incubation	Sáenz et al. (2019)
*Aspergillus terreus* MF12	Plastic waste dump yard	India	Physically and chemically pretreated HDPE	Weight loss, structural and morphological changes after 30 d of incubation	Balasubramanian et al. (2014)
*Cephalosporium* sp.	Culture	India	Nitric acid-pretreated HDPE films	Weight loss, decrease in pH of liquid culture media, increase of TDS and conductivity, chemical and morphological changes after 20 d of incubation	Chaudhary and Vijayakumar (2020a)
*Penicillium chrysogenum* NS10 (KU559907), *Penicillium oxalicum* NS4 (KU559906)	Soil	India	HDPE and LDPE films	Weight loss, morphological damages on PE sheets after 60 d of growth	Ojha et al. (2017)
*Alternaria alternata, Aspergillus caespitosus, Aspergillus terreus, Eupenicillium hirayamae*, *Phialophora alba, Paecilomyces variotii*	Mangrove of Red Sea coast	Saudi Arabia	LDPE film	Higher biomass, CO_2_ release, production of ligninolytic enzymes	Ameen et al. (2015)
*Aspergillus clavatus* JASK1	Soil	India	LDPE	Weight loss, morphological changes, CO_2_ release after 90 d of incubation	Gajendiran et al. (2016)
*Aspergillus flavus*, *Aspergillus niger*	Garbage soil	India	LDPE granules	Weight loss, morphological changes after 6 months of incubation	Deepika and Jaya (2015)
*Aspergillus flavus*, *Aspergillus japonicas*, *Aspergillus niger*, *Fusarium* sp., *Penicillium* sp., *Mucor* sp.	Plastic contaminated soil	India	LDPE sheets	Weight loss, increased fresh weight of fungi after 4 weeks of incubation	Singh and Gupta (2014)
*Aspergillus flavus*, *Aspergillus terreus*	Landfill soil	India	LDPE in soil and synthetic media	Weight loss, morphological and chemical changes after 4 and 9 months incubation in soil and media respectively	Verma and Gupta (2019)
*Aspergillus flavus*, *Mucor rouxii* NRRL 1835	Culture	Egypt	Plastic strips	Increase in production of reducing sugar and protein in culture supernatant, decrease in tensile strength after 1 month of incubation at 30 °C	El-Shafei et al. (1998)
*Aspergillus fumigatus, Aspergillus terreus*	Landfill soil	Iran	Photo-oxidized LDPE films	Reduction in total organic carbon and Mw, morphological and chemical changes	Zahra et al. (2010)
*Aspergillus fumigatus* L30, *Aspergillus terreus* HC	Soil	Taiwan	PBSA	Fungal colonization, weight loss, morphological and chemical changes after 30 d of incubation, synthesis of lipolytic enzymes	Chien et al. (2022)
*Aspergillus fumigatus*, *Xylaria* sp.	Landfill soil	India	LDPE strips	Morphological changes, CO_2_ release, pH change within 30 d incubation	Jeeva Dharshni and Kanchana (2021)
*Aspergillus fumigatus* B2,2(MG779513), *Aspergillus nidulans* E1,2 (MG779511), *Aspergillus oryzae* A5,1(MG779508)	Plastic dumpsite	Kenya	LDPE	Weight loss, chemical change after 16 weeks of incubation	Muhonja et al. (2018)
*Aspergillus niger*, *Aspergillus japonicas*	Polythene bags from polluted site	India	LDPE strips	Weight loss, morphological changes after 1 month of incubation	Raaman et al. (2012)
*Aspergillus niger*, *Penicillium pinophilum*	Culture	Mexico	Thermo-oxidized and ethanol treated LDPE	Decrease in crystanility, morphological and chemical changes, mineralization of the polymer after 31 months of incubation	Volke-Sepúlveda et al. (2002)
*Aspergillus ustus*, *Penicillium verrucosum*	Soil	Poland	PLA foil	Increase in mycelial growth, structural and thermal changes of the foil after 10 d incubation at 30 °C	Szumigaj et al. (2008)
*Aspergillus nomius, Trichoderma viride*	Landfill soil	Indonesia	LDPE films	Weight loss, reduction in tensile strength, morphological changes after 45 d of growth	Munir et al. (2018)
*Aspergillus versicolor*	Sea water	India	LDPE	Increased fresh weight of fungi, CO_2_ release	Pramila and Vijaya Ramesh (2011)
*Aspergillus* sp., *Paecilomyces lilacinus*	Leaf and stem of *Humboldtia brunonis*	India	Irradiated LDPE	Weight loss, formed biofilm	Sheik et al. (2015)
*Aspergillus* sp., *Fusarium* sp.	Soil buried LDPE films	India	LDPE films	Weight loss, reduction in pH, CO_2_ release, structural and morphological changes over a period of 60 d	Das and Kumar (2014)
*Aspergillus* sp., *Penicillium* sp.	NM	Brazil	PP/PBAT/thermoplastic starch blends	Chemical and morphological changes after 1 month incubation	de Oliveira et al. (2020a; 2020b)
*Cladosporium cladosporioides*, *Purpureocillium lilacinum*	Agricultural soil	Thailand	PLA, PBS, PCL	Clear zones formation on agar plates	Penkhrue et al. (2015)
*Diaporthe italiana, Thyrostroma jaczewskii, Stagonosporopsis citrulli*	Culture	Thailand	LDPE MP	Weight loss, reduction in tensile strength, chemical and morphological changes, CO_2_ release within 90 d of incubation; production of laccase, MnP and LiP	Khruengsai et al. (2021)
*Fusarium falciforme*, *Fusarium oxysporum, Purpureocillum lilacinum*	Landfill soil	Italy	LDPE	Biofilm formation, morphological changes, CO_2_ release	Spina et al. (2021)
*Fusarium solani* VKM F-4202	Culture	Russian Federation	PCL	Sample degradation within 30 d of cultivation	Antipova et al. (2018)
*Aspergillus calidoustus* VKM F-2909, *Parengyodontium album* VKM F-3028	Culture	Russian Federation	PLA	Sample degradation within 30 d of cultivation	Antipova et al. (2018)
*Fusarium* sp.	Soil	Pakistan	UV irradiated and nitric acid treated LDPE	Biomass increase, chemical change after 3 months of incubation	Hasan et al. (2007)
*Penicillium simplicissimum* Bar2	Waste plastic carry bag	India	Alcohol pretreated LDPE sheet	Weight loss, morphological and chemical change, CO_2_ release, lipase production	Ghosh and Pal (2021)
*Penicillium simplicissimum*	Dumpsite soil	India	Autoclaved, UV-treated and surface-sterilized PE	Weight loss, morphological and chemical change after 3 months incubation, laccase and MnP production	Sowmya et al. (2015)
*Paecilomyces lilacinus* D218	Soil	Japan	PHB, PCL	Degraded polymers within 10 d incubation, depolymerase production	Oda et al. (1995)
*Rhizopus oryzae* NS5 (Accession No. KT160362)	Culture	India	Thermally treated LDPE film	Weight loss, reduction in tensile strength, decrease of pH and contact angel, morphological and structural changes of PE after 30 d of incubation	Awasthi et al. (2017)
*Thermomyces lanuginosus*	Culture	India	UV, thermally, and chemically pretreated LDPE	Weight loss, chemical change after 1 month of incubation	Chaudhary et al. (2021)
Mixed Culture of *Lysinibacillus xylanilyticus* and *Aspergillus niger*	Landfill soil	Iran	UV irradiated LDPE films	Structural and morphological changes of plastics, CO_2_ release	Esmaeili et al. (2013)
*Alternaria* sp.	Bath tub	Japan	Plasticized PVC rim	Morphological change	Moriyama et al. (1993)
*Aspergillus niger* PV3, *Aspergillus sydowii* PV4, *Lentinus tigrinus* PV2, *Phanerochaete chrysosporium* PV1	Soil	Pakistan	PVC film	Structural and morphological changes, Mw reduction, CO_2_ release after 10 months of incubation	Ali et al. (2014b)
*Aureobasidium pullulans*	Atmosphere	United Kingdom	Plasticized PVC film	Degraded plasticizer DOA, weight loss of PVC, esterase production	Webb et al. (2000)
*Chaetomium globosum* (ATCC 16,021)	Culture	Brazil	PVC	Weight loss, morphological changes, growth of perithecia after 28 d	Vivi et al. (2019)
*Cochliobolus* sp.	Landfill soil	India	PVC	Morphological and structural changes after 3 months of incubation; laccase production	Sumathi et al. (2016)
*Penicillium janthinellum*	PVC buried in soil	United Kingdom	Plasticized PVC sheet	Weight loss, change in physical properties after 10 months of incubation	Sabev et al. (2006)
*Phanerocheate chrysosporium*	Plastic disposal site	Pakistan	PVC film	Weight loss, structural and morphological changes, LiP production	Khatoon et al. (2019)
*Aspergillus* sp., *Penicillium* sp.	NM	Brazil	PP	Structural and morphological changes after 30 d incubation	Oliveira et al. (2020a, b)
*Aspergillus fumigatus*	Soil	India	PP cup	Weight loss, structural and morphological changes after 6 months of incubation	Oliya et al. (2020)
*Bjerkandera adusta*	Culture	Romania	Gamma irradiated PP	Structural and morphological changes after 7 weeks of incubation	Butnaru et al. (2016)
*Yarrowia lipolytica* 78 − 003	Culture	USA	Thermally depolymerized PP	Structural change	Mihreteab et al. (2019)
*Yarrowia lipolytica* IMUFRJ 50,682	Guanabara Bay estuary	Brazil	PET		da Costa et al. (2020)
*Cephalosporium* sp., *Mucor* sp.	Culture	India	PS foam	Reduction of pH, Mw and thermal stability; increase in TDS and conductivity; weight loss, morphological changes after 8 weeks of incubation	Chaudhary and Vijayakumar (2020b)
*Ceriporia* sp. BIOM3, *Cymatoderma dendriticum* WM01, *Pestalotiopsis* sp. NG007	NM	Indonesia	Styrofoam	Weight loss, morphological and structural changes within 30 d of incubation	Yanto et al. (2019)
*Agaricus bisporus*, *Marasmius oreades*	Fruiting body	Switzerland	PU	Formed halos in agar medium containing PU powder	Brunner et al. (2018)
*Alternaria tenuissima*	NM	Romania	Pyridine-based polyether PU film	Structural and morphological changes, loss of tensile strength	Oprea et al. (2018)
*Alternaria* sp., *Aspergillus* sp., *Penicillium* sp.	Multiple collection	France	Impranil	Used PPU as the sole carbon source	Magnin et al. (2019)
*Alternaria* sp.	Environment	Japan	Ether type PU film	Weight loss, structural and morphological changes	Matsumiya et al. (2010)
*Aspergillus niger, Cladosporium herbarum*	Culture	Germany	PU foam	Morphological and structural changes after 70 d	Filip (1979)
*Aspergillus tubingensis*	Soil from waste disposal site	Pakistan	Polyester PU film	Mycelial colonization, morphological and chemical changes, loss of tensile strength, degradation of the film in small pieces after 2 months in liquid medium	Khan et al. (2017)
*Aspergillus* sp.	Soil from waste-dumping site	Pakistan	Polyester PU film	Weight loss, chemical and morphological changes, CO_2_ release, increase in melting temperature, esterase production	Osman et al. (2018)
*Aureobasidium pullulans*, *Cladosporium* sp., *Curvularia senegalensis*, *Fusarium solani*	Garden soil	USA	PU	Zone of clearance was observed	Crabbe et al. (1994)
*Cladosporium cladosporioides*, *Leptosphaeria* sp., *Penicillium griseofulvum*, *Xepiculopsis graminea*	Plastic debris	Switzerland	PU	Formed halos in agar medium containing PU powder	Brunner et al. (2018)
*Fusarium solani* H14	Contaminated soil	China	PU film	Mass reduction, morphological changes of PU within 90 s incubation, lipase and esterase production	Ren et al. (2021)
*Geomyces pannorum*	Acidic soil	United Kingdom	PU, Impranil	Loss of tensile strength of PU after 5 months of soil burial, degraded Impranil	Cosgrove et al. (2007)
*Microsphaeropsis arundinis* CBMAI 2109, CBMAI 2110	Fresh water	Brazil	PET	Weight loss, morphological and chemical changes, lipase and esterase production after 14 d of incubation	Malafatti-Picca et al. (2019)
*Monascus* sp.	Plastic contaminated soil	Egypt	PU (Impranil DLN)	Cleared PU in liquid medium, increased zeta potential, morphology change after 14 d, esterase production	El-Morsy et al. (2017)
*Pestalotiopsis microspora*	Plant substrate	Switzerland	PU	Formed halos in agar medium containing PU powder	Brunner et al. (2018)
*Pestalotiopsis microspora*	Plant stem	Ecuador	Impranil DLN	Increase in fungal biomass, chemical change	Russell et al. (2011)
*Phoma* sp.	Neutral soil	United Kingdom	PU, Impranil	Loss of tensile strength of PU after 5 months of soil burial, degraded Impranil	Cosgrove et al. (2007)
*Phanerochaete chrysosporium* (ATCC 34,541)	Culture	Turkey	LDPE/starch blend film	Increase in biomass, reduction in relative viscosity, CO_2_ release	Orhan and Büyükgüngör (2000)
*Lasiodiplodia theobromae*	Leaf and stem of *Psychotria flavida*	India	Irradiated LDPE, PP	Weight loss, biofilm formation	Sheik et al. (2015)
*Phanerochaete chrysosporium* PV1	Soil	Pakistan	Starch blended PVC	Mw reduction, morphological and chemical changes of plastic, CO_2_ release	Ali et al. (2014a)
*Pleurotus ostreatus* PLO6	Culture	Brazil	Sunlight exposed GP	Mechanical and structural changes, CO_2_ release after 30 d incubation	da Luz et al. (2015)
*Pleurotus ostreatus* PLO6	Culture	Brazil	Sunlight exposed oxo-biodegradable polymers	Reduction in dry mass of substrates	da Luz et al. (2014)
*Pleurotus ostreatus* PLO6	Culture	Brazil	Oxo-biodegradable plastic	Chemical and morphological changes after 46 d incubation	da Luz et al. (2013)
*Bjerkandera adusta*	Factory producing nylon-6	Slovenia	Nylon fiber	Weight loss, decrease in viscosity, changes in morphological and thermal properties, molecular mass reduction	Friedrich et al. (2007)
*Fusarium* sp.	Composted soil	Japan	Nylon 4 film	Structural and morphological changes after 2 months of incubation	Tachibana et al. (2010)
*Fusarium* sp. B30M, *Hansenula anomala*, *Sclerotinia* sp. B11IV	Soil from Antarctica	Poland	PBSA, PCL	Plastic degradation in shake-flask cultures, morphological changes within one moth of incubation	Urbanek et al. (2021)
*Aspergillus oryzae*	Plastic contaminated soil	India	Plastic	Morphological changes, loss of weight within 60 d of incubation	Indumathi and Gayathri (2016)
*Geomyces* spp., *Mortierella* spp., *Penicillium* spp.	Soil	Antartica	UV treated PE, PS, PU	Weight loss, chemical structures changes after 90 d of incubation	Oviedo-Anchundia et al. (2021)
*Aspergillus fumigatus*, *Aspergillus niger*, *Fusarium oxysporum*, *Penicillium* sp.	Landfill soil	India	LDPE and PU sheets	Zone of clearance in growth media, weight loss, decrese in tensile strength within 90 d culture	Raghavendra et al. (2016)
*Pseudozyma antarctica* JCM 10,317	Leaves and husks of paddy rice	Japan	PBS and PBSA mulch films	Degraded both PBs and PBSA, biodegradable plastic-degrading enzyme production	Kitamoto et al. (2011)
*Penicillium* sp.	Plastic waste dumped soil	India	PET powder and flakes	Structural and morphological changes; fulgal colonization after 4 weeks	Sepperumal et al. (2013)
*Penicillium funiculosum*	Dumped site	Poland	PET films	Weight loss, chemical and morphological changes after 84 d	Nowak et al. (2011)
*Penicillium oxalicum* SS2	Soil	Pakistan	PHB, PHBV	Degradation of BPs emulsion and film within 48 h, morphological and chemical changes	Satti et al. (2020)
*Penicillium oxalicum* DSYD05-1	UV mutant of wild type	China	PCL	Weight loss, morphological and chemical changes	Li et al. (2012)
*Penicillium variabile* CCF3219	NM	China	Ozone-pretreated [β-^14^ C]-PS film	Mw reduction, chemical and morphological changes after 16 weeks incubation	Tian et al. (2017)
*Pestalotiopsis* sp.	*Nepenthes ampullaria*	Malaysia	PU	Zone of clearance in medium after 2–3 weeks of incubation	Lii et al. (2017)
*Clonostachys rosea*, *Trichoderma* sp.	Arctic soil	Norway	PCL	Weight loss, morphological changes	Urbanek et al. (2017)
*Candida tropicalis*	Culture	Malaysia	LDPE, SBP	Weight loss, morphological changes, biofilm formation	Zahari et al. (2021)
*Trichoderma viride* GZ1	NM	Poland	PLA, PET	Hydrophobin production, chemical and morphological changes after 3 months of incubation	Dąbrowska et al. (2021)
*Absidia* sp., *Actinomucor elegans*, *Aspergillus fumigatus, Aureobasidium pullulans, Bjerkandera adusta, Cladosporium subcinereum, Fusarium* sp., *Mortierella* sp., *Penicillium* sp., *Trichoderma harzianum*	Agricultural soil	Slovakia	PLA, PHB and PLA/PHB blend polymers	Weight loss after 1 year of mineralization	Jeszeová et al. (2018)
*Engyodontium album* MTP091 (SF1), *Pencillium* sp. MTP093 (SF3), *Phanerochaete chrysosporium* NCIM 1170 (SF2)	Soil (*E. album*, *Pencillium* sp.), culture (*P. chrysosporium*)	India	Bisphenol A polycarbonate	Weight loss, reduction in Mw, structural changes	Artham and Doble (2010)

*DOA* dioctyl phthalate and dioctyl adipate, *GP* green polythene, *NM* not mentioned

Pretreatment has also been found to be an important tool for HDPE degradation by fungi as reported in earlier studies (Supplementary Fig. 3). For instance, exposure of *Cephalosporium* sp. caused 7.18% weight loss of HDPE polymer, pretreated with nitric acid, after 20 days of incubation period. Moreover, decrease in crystallinity and pH of liquid culture media as well as increase in total dissolved solid (TDS) and conductivity further confirmed ability of the fungus to utilize HDPE for growth (Chaudhary and Vijayakumar 2020a). Earlier, Mathur et al. (2011) investigated on ability of *Aspergillus niger* to degrade HDPE that was subjected to thermal treatment by placing the plastic at 70 °C for 10 days. After 1 month incubation, heavy colonization, hyphal penetration in the film, reduction in mass (3.44%) and tensile strength (61%) of PE were observed. Further analyses showed cracks on the surface of polymer and decrease in amount of carbonyl residues indicating great possibility of the fungus to degrade HDPE. In contrast, some studies have shown that fungi can break down HDPE without any prior treatments. As an example, incubation of *Aspergillus tubingensis* and *Aspergillus flavus* with HDPE for 1 month resulted 6.02 and 8.51% weight loss of the plastic respectively signifying higher efficiency of *A. flavus*. Moreover, biofilm formation, changes in surface topography of the film and chemical alterations such as decrease in carbonyl index were also recorded. Experiment was also performed to enquire effect of mineral oil on the colonization efficacy where results suggested that presence of the light white oil stimulated hydrophobic interaction between the taxa and polymer surface promoting rate of biodegradation (Devi et al. 2015). In another work, *A. flavus* isolated from gut contents of wax moth *Galleria mellonella*, have been reported to degrade HDPE MPs into particles of low Mw after 28 d of incubation where the process was might be triggered by laccase-like multicopper oxidases (Zhang et al. 2020). Thus, it could be predicted that fungi are gradually adapting toward HDPE biodegradation by natural evolution.

In respect to LDPE bio-degradation, the genus *Aspergillus* has emerged as an efficient organism where Muhonja et al. (2018) attributed the effect to *Aspergillus oryzae*. They have also elucidated fungi as better degraders of PE in comparison to bacteria which could be due to higher production of laccase and esterase. In another study, *Trichoderma viride* and *Aspergillus nomius* were found as potent plastic degrader as they reduced weight of the LDPE film by 5.13% and 6.63% respectively after 45 d of cultivation in mineral salt agar medium supplemented with 0.5% glucose. While, tensile strength of the treated films was reduced significantly by 58% and 40%. Analyses of electron micrograph exhibited formation of grove and rough on the surface of LDPE. Comparatively, the outcome was found to be lower than that of *Aspergillus japonicus* and *A. niger* as they showed 12% and 8% LDPE degradation ability respectively under laboratory conditions within 1 month period. SEM analysis further confirmed the efficacy as evident by fragility and presence of porosity on the fungal degraded PE surface (Raaman et al. 2012). *Aspergillus niger* and *A. terreus* were isolated from a mangrove in Ecuador and they degraded up to 35.3% and 22.14% of LDPE films weight over a period of 77 days (Sáenz et al. 2019). However the outcome was comparatively lower than that of *Aspergillus clavatus* as the fungus caused 35% weight loss of LDPE films after 2 months of incubation. Further, 2.32 g/l CO_2_ production was found when the specimen was incubated with the plastic for 1 month (Gajendiran et al. 2016). The findings are similar with Shah et al. (2009) reporting 1.85 g/l CO_2_ evolution after 1 month period of incubation with *Fusarium* sp. on LDPE films. The Ascomycetes was also capable of adhering to the PE surface manifested by increase in mycelial growth, when cultured in basal salts medium at 30 °C and 150 rpm for 2 months. Further, morphological changes in PE pieces like appearance of pits, cracks and erosion were detected as well indicating ability of the fungus of utilizing PE as the source of carbon and energy. Earlier, an extensive study has been reported by Ameen et al. (2015) that was designed to isolate potent LDPE degrading fungal isolates from the marine water. At per the results, six isolates were able to grow profusely on the film along with production of more ligninolytic enzymes and release of CO_2_. The overall effect was found to be in the decreasing order of *Alternaria alternata* > *A. terreus* > *Eupenicillium hirayamae* > *Paecilomyces variotii* > *Phialophora alba* and *Aspergillus caespitosus*. Interestingly, consortium of all these isolates portrayed the best degradation effect suggesting a synergistic consequence of the fungi.

In contrast to previous findings, few studies have reported on fungal degradation of LDPE pretreated in various ways. For instance, Sheik et al. (2015) irradiated PE with different doses of radiation (0–1000 kGy) and incubated with endophytic fungi for 90 days. The specimens namely *Aspergillus* sp., *Paecilomyces lilacinus* and *Lasiodiplodia theobromae* caused decrease in intrinsic viscosity and average Mw of the LDPE strips. Further, the fungi were able to grow profusely over hydrophobic surface of plastic films which might be aided by laccase. In another study, *Fusarium* sp. was found to grow better in mineral salt medium with LDPE pieces pretreated with UV for about 250 h followed by nitric acid at 80 °C for 6 days (Hasan et al. 2007) suggesting combination of physical pretreatment with myco-remediation could significantly enhance the overall efficiency of the process.

### Effect of fungal activity on PVC

Till date, only few studies have been published identifying fungal strains with PVC degradable ability. Ali et al. (2014b) performed soil burial experiment and asserted *P. chrysosporium*, *L. tigrinus*, *A. niger* and *A. sydowii* to flourish on PVC film after 10 months of incubation. The findings were further verified by shake flask method where *P. chrysosporium* caused maximum biomass production, better CO_2_ release (7.85 g/l) and reduction in Mw of the film by about 1 kDa. The same fungus has been used in another study where it exhibited better potency in degradation of starch blended PVC. As such, the incubation for 1 month resulted decrase of Mw of the polymer by around 2 kDa along with 7.31 g/l CO_2_ release (Ali et al. 2014a). These observations are substantially in line with other studies where researchers treated cellulose blended PVC with cellulolytic fungi like *Trichocladium* sp. and *Chaetomium* sp. (Kaczmarek and Bajer 2007). So far, only few studies have documented the participation of fungal ligninolytic enzymes in PVC biodegradation (Khatoon et al. 2019); however effort on quantification and purification of the enzyme is limited.

### Effect of fungal activity on PP

Presence of methyl group in β position makes PP a hardly biodegradable plastic, particularly in absence of pretreatment processes. Therefore, finding potent microorganisms with PP deterioration activity is highly required; though very few studies have been reported till date. Sheik et al. (2015) designed an experiment where PP films were irradiated at different doses of gamma radiation ranging from 0 to 100 kGy to understand effect of pretreatment on the polymer degradation by fungi. Amongst the investigated species, only *L. theobromae* inoculated film showed weight loss after 3 months of incubation where the effect was more prominent from 40 kGy dose onwards. Further, a discernible decrease in Mw and viscosity of the PP films was also recorded. FT-IR data revealed that carbonyl groups appeared from 60 to 100 kGy in gamma irradiated film indicating that higher doses can increase sensitivity of plastics towards the fungi. In a separate study, *A. fumigatus* has been reported to degrade up to 18.08% of PP over a period of 6 months (Oliya et al. 2020). To date, there has been no investigation into the utilization of fungal enzymes for the biodegradation of PP at per our knowledge.

### Effect of fungal activity on PS

Reports of PS destruction in nature are scarce and typically indicate only little degradation. However, fungi could represent promising candidates for PS biodegradation due to their numerous unspecific and powerful extracellular oxidation mechanisms (Krueger et al. 2017). Tian et al. (2017) synthesized two different types of ^14 ^C-labelled PS polymers and investigated effect of *Penicillium variabile* CCF3219 as well as ozonation on the mineralisation process. The results showed that the specimen mineralised both labelled polymers, and the [U-ring-^14^ C]-PS with a lower Mw led to a higher mineralisation rate. GPC analysis further showed that Mw of the ozonated [β-^14^ C]-PS decreased after incubation. The investigation hence suggests that the pretreatment could be a potential approach for PS waste degradation and remediation of plastic-contaminated locations. Recently Chaudhary and Vijayakumar (2020b) reported that incubation of *Cephalosporium* sp. and *Mucor* sp. with PS for 2 months resulted a weight loss of 2.17% and 1.81% respectively. The same time duration has also been used by Oviedo-Anchundia et al. (2021) reporting 8.39% and 6.82% PS degradation abilities of *Penicillium* sp. and *Geomyces* sp. respectively; in contrast to that *Mortierella* sp. degraded the polymeric material only by 2.19%. Further, it was inferred that the investigated fungi were more active on aged polymer, possibly due to the molecular alterations induced by pretreatment with UV radiation.

### Effect of fungal activity on PU

Recent research has portrayed that PU in acidic and neutral soil is susceptible to microbial attacks where fungi can act as the predominant degrading microbes (Cosgrove et al. 2007). Various studies across the globe have conveyed potent PU degrading fungi isolated from sand, dumping areas, contaminated soils, compost, plastic waste, wall paint and plastic debris floating near lakeshores (Ren et al. 2021). Several types of PU materials such as polyether urethane foam, thermoplastic polyester and PU waterborne dispersion of polyester PU have been appeared as sensitive to fungal degradation. One example is the research of Cosgrove et al. (2007) who reported on the fungal communities associated with in situ degradation of polyester PU buried in two sandy loam soils for 5 months. *Phoma* sp. was identified as the main cultivable organism collected from the PU surface buried in neutral soil (pH 6.7). Whilst, PU buried in acidic soil (pH 5.5) was found to be dominated by *Geomyces pannorum* and *Nectria* sp. indicating that the soil type plays an important role on the composition of plastic degrading fungal communities. Both the fungi represented >80% of cultivable colonies from each plastic and degraded Impranil (an anionic and aromatic polyether-PU dispersion) also. In a previous study, *Curvularia senegalensis* was observed to possess better PU-degrading activity and further analysis lead to purification of an extracellular polyurethanase displaying esterase activity (Crabbe et al. 1994). Esterase production has also been found in *Monascus ruber*, *Monascus sanguineus* and *Monascus* sp. where the researchers indicated a correlation between enzyme synthesis by the fungal strains and sampling sites (El-Morsy et al. 2017). Russell et al. (2011) isolated endophytes from Ecuadorian Amazonian plants to screen for their ability to degrade PU where all active fungi were found to belong to Ascomycota. Amongst them, *Pestalotiopsis microspora* was identified as the better PU degrader under both aerobic and anaerobic conditions. The endophytic fungus appeared to produce extracellular and diffusible serine hydrolase responsible for PU degradation. Putative polyurethanases have been isolated from protein extracts of *Candida rugosa* as well with reaction optima of pH 7 and 35 °C which may help to develop bioreactors for real-time application to manage PU trash (Gautam et al. 2007). Recently, *F. solani* was screened from soil contaminated with explosive rocket propellant material. Results indicated that the fungus was able to cause 25.8% mass loss for PU after 2 months along with morphological changes. Two possible degradation enzymes, namely lipase and esterase, were found to be synthesized by the specimen (Ren et al. 2021). The same enzymes have also been detected in *A. tubingensis* that was able to grow on the surface of PU film degrading the polymer (Khan et al. 2017). Additional studies identified *F. solani* and *Candida ethanolica* as PU degraders (Zafar et al. 2013); although associated enzymes have not yet been identified from them.

### Effect of fungal activity on polyamide (PA) or nylon

PA is generally considered as non-biodegradable polymers; but recent studies have enumerated metabolism of PA by numerous microbes. Friedrich et al. (2007) conducted research on several fungi for their ability to degrade nylon fibres. Amongst them, *Bjerkandera adusta* disintegrated the fibres most efficiently after incubation for several weeks. MnP was detected in the liquid phase and presumed to be responsible for the degradation. The observation was in accord to Deguchi et al. (1998) as they found a nylon-degrading enzyme of Mw of 43 kDa in the extracellular medium of a ligninolytic culture of a white rot fungus. The protein was further purified that showed identical characteristics to those of MnP; however the activity of the native and purified enzyme varied from that of lignolytic enzyme. Structural degeneration of the nylon-66 membranes further confirmed the polymer-degrading activity. In a separate study, nylon 4 films were immersed in the broths containing minerals and *Fusarium* sp. As a result, weight of the films was found to decrease and disappear within 2 months. However, isolation and characterization of the associated enzyme remained elusive (Tachibana et al. 2010).

### Effect of fungal activity on biodegradable plastics (BPs)

**Fig. 3:**
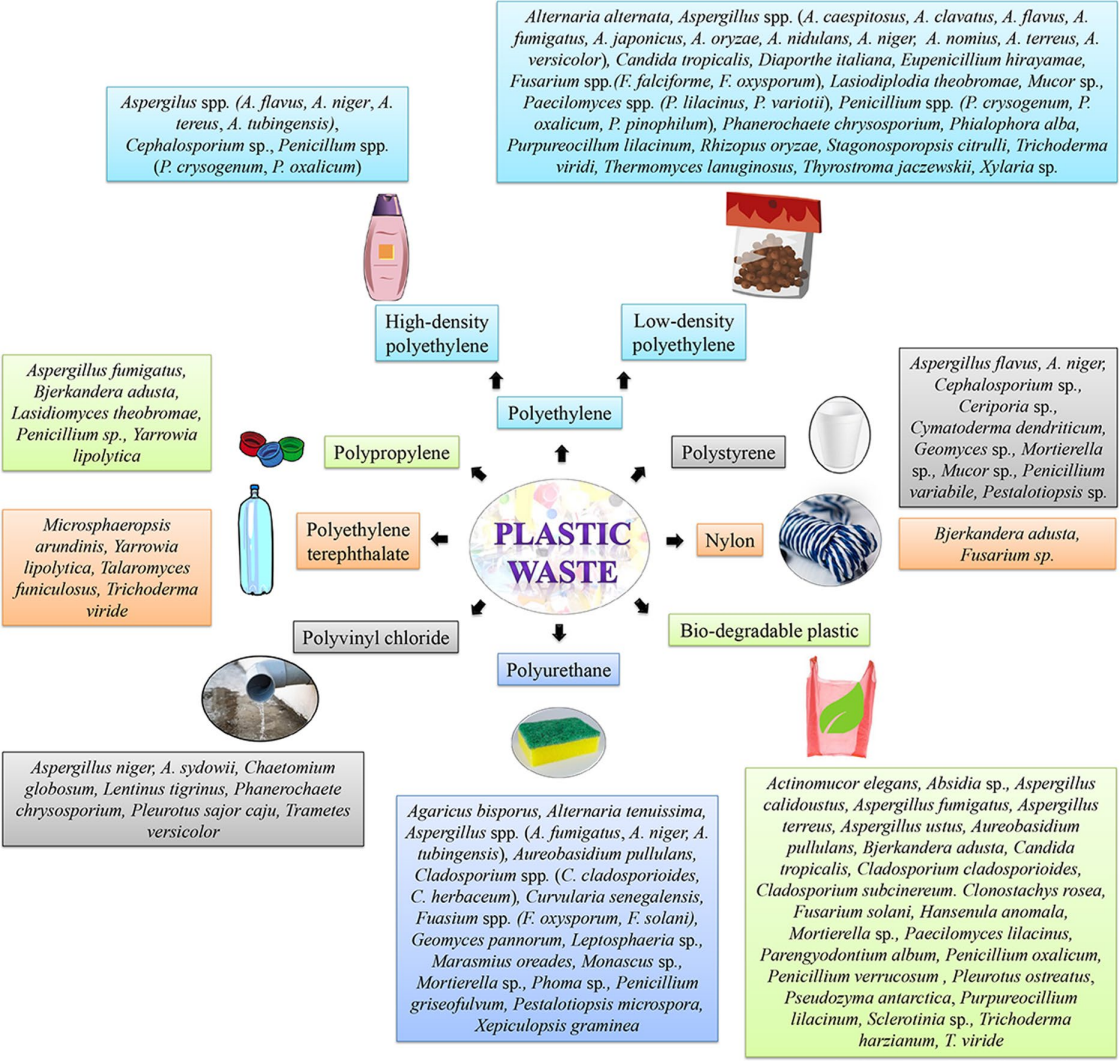
Effect of different fungi on various types of plastics as published earlier

Recently, many researchers have confirmed that fungi have great potential to degrade several BPs including poly(lactic acid) (PLA) (Torres et al. 1996) and poly (L-lactide) (PLLA) (Jarerat and Tokiwa 2001) (Fig. 3). It showed that most of PLA film was degraded after 14 days of cultivation at 30 °C by the addition of gelatin. Some researchers have also explored ability of fungi for degradation of PLA in soil and compost (Karamanlioglu et al. 2013; Saadi et al. 2012). The research depicted temperature as a key parameter governing the fungal degradation of PLA. In addition, Lipsa et al. (2016) assessed biodegradation of PLA and some plasticized PLA with *Trichoderma viride* in liquid medium and controlled laboratory conditions. Degradation of low-Mw PLA has also been investigated with lipase from *Rhizopus delemer* (Fukuzaki et al. 1989). In contrast to PLA, several studies have been carried out to evaluate the biodegradation of poly(ε-caprolactone) (PCL). Tokiwa et al. (1976) isolated *Penicillium* sp. utilizing PCL for its growth and found PCL depolymerase activity in extracellular and intracellular fractions. The break down of PCL has also been manifested by lipase of *Candida antarctica* and cutinase of *F. solani* (Shi et al. 2020). In a recent study, Kosiorowska et al. (2021) aimed for co-expression of heterologous cutinase and native lipase in *Y. lipolytica* that resulted efficient degradation of PCL at 28 °C.

Poly(butylene succinate) (PBS) and its copolymers poly(butylene succinate-co-adipate) (PBSA) are comparatively recent and their fungal degradation has not been studied in detail. For instance, Li et al. (2011) prepared a UV mutant *Aspergillus* sp. XH0501-a that exhibited 38.89% better PBS degradation ability than that of wild-type strain. Further, a novel extracellular PBS-degrading enzyme with Mw of 44.7 kDa was purified. The optimum temperature and pH for the enzyme activity was 40 °C and 8.6 respectively. It was found that Fe^2+^ and Ca^2+^ enhanced the function, whereas Cu^2+^ and Hg^2+^ inhibited it. Chien et al. (2022) selected two elite PBSA-degrading *Aspergillus* strains, namely, *A. terreus* HC and *A. fumigatus* L30, from soil samples in Taiwan and incubated in carbon-free basal medium. Results showed that *A. fumigatus* and *A. terreus* deteriorated PBSA films by about 26% and 42% respectively within 1 month. In the soil burial test, *A. terreus* exhibited more than 90% and 75% degradation rates for summer and winter soil respectively; where further adding of mycelia to the winter soil, improved the degradation efficacy of PBSA. Further, the process of degradation or inclusion of fungal mycelia did not cause any adverse effect on growth of Chinese cabbage indicating non-toxic effect of the method. Kitamoto et al. (2011) reported potency of various strains of *Pseudozyma* and *Cryptococcus* to degrade PBS and PBSA films. Further, approximately a 22 kDa plastic-degrading enzyme was found to be secreted by the type strain, *P. antarctica* and *Pseudozyma* spp. In another approach, *P. antarctica* exhibited a strong degradation activity for BPs such as agricultural mulch films. An enzyme consisting of 198 amino acids was further isolated that degraded solid films of PBSA, PBS, PCL and PLA (Shinozaki et al. 2013b).

Indeed, it has been observed that several fungi have the ability to decompose more than one type of bioplastic, for instance, Urbanek et al. (2017) found eight fungal strains as PLA, PBS, PBSA or PCL degraders among 24 tested strains. Further, these strains were identified as species of *Clonostachys rosea* and *Trichoderma* that exhibited PCL degradation by 52.91% and 21.54% after 1 month incubation at 28 °C respectively. Penkhrue et al. (2015) described that *C. cladosporioides* and *Purpureocillium lilacinum* could form clear zones on PLA, PBS and PCL agar plates. This could be due to capcity of the fungi to excrete enzymes that can diffuse through agar and degrade the polymer into water soluble materials. Antipova et al. (2018) found that *F. solani* VKM F-4202 that could degrade about 90% of PCL within 30 days of cultivation. During the process, a culture medium containing peptones was selected, that facilitated synthesis of depolymerases decomposing PLA. On the other hand, *Parengyodontium album* VKM F-3028 and *Aspergillus calidoustus* VKM F-2909 efficiently degraded PLA samples within 1 month of cultivation. Jeszeová et al. (2018) investigated on the microbial communities responsible for degradation of PLA/PHB blend foils and for that they performed 1 year long laboratory soil burial experiments. Several PLA/PHB blend degrading microorganisms were isolated belonging to the genera *Aureobasidium*, *Mortierella*, *Fusarium*, *Absidia*, *Bjerkandera*, *Actinomucor*, *Trichoderma* and *Penicillium*. Sameshima-Yamashita et al. (2016) isolated an enzyme from *Paraphoma* like fungus strain B47-9 that could degrade commercial biodegradable films within eight h. Paraphoma-related fungal strain B47-9 secreted a BP-degrading enzyme belonging to the cutinase family; thus, it was named Paraphomarelated fungus cutinase-like enzyme (PCLE). It degraded various types of BP films, such as PBS, PBSA, poly(butylene adipate-co-terephthalate), PCL, and poly(DL-lactic acid). It has a molecular mass of 19.7 kDa, and an optimum pH and temperature for degradation of emulsified PBSA of 7.2 and 45 °C, respectively (Suzuki et al. 2014). Shinozaki et al. (2013a, b) described that esterases from *Pseudozyma antarctica* (PaE) was able to degrade BPs. The degradation profiles of plastic films composed of poly(butylene succinate), poly(butylene succinate-co-adipate), or poly(-butylene adipate) by these enzymes as charcterized by liquid chromatography-mass spectrometry and size exclusion chromatography (Sato et al. 2017). Oda et al. (1995) isolated five fungi from soil and amongst them, *P. lilacinus* was identified as efficient degrader of BPs as evident by formation of of 3-hydroxybutyrate from PHB and ɛ-caprolactone from PCL after incubation. The fungus was also found to excret PHB and PCL depolymerases in media containing either PHB, PCL or PHB plus PCL. A thermotolerant *Aspergillus* sp. ST-01 degraded more than 90% film samples of PHB and PCL at 50 °C. The degradation products were identified as succinic acid, butyric acid, valeric acid, and caproic acid (Sanchez et al. 2000). Overall, it was found that PHB and PCL-degrading microbes possess broader distribution in a wide range of environments than PLA degraders. Sankhla et al. (2020) enumerated distribution profile of the whole polymer-degrading microbes in soils as an order of PCL = PHB > PBS > PLA.

Sometimes, pro-oxidants (e.g. metal ions or oxides) and pro-degrading substances are included into the polymeric chain to augment photo- or thermo-oxidation and such polymers are called oxo-biodegradable polymers. The pro-oxidants helps in production of free radicals during photo-degradation, also termed pro-oxidant photocatalytic oxidation, resulting scission in the polymer chain promoting microbial degradation (Kitamoto et al. 2011). Till date, only few fungi have been reported to possess striking capacity to deteriorate such BPs. For instance, *P. ostreatus* has been found to form biofilm and break down oxo-BPs deprived of any previous physical treatment. The oxidation was suspected to be mainly due to laccase activity (da Luz et al. 2013). As a follow up study, the mushroom was subjected to oxo-BPs bags that were exposed to sunlight for up to 120 days. Results showed that *P. ostreatus* was able to grow using the plastics as a source of carbon and energy (da Luz et al. 2014). On oxo-biodegradable PE samples kept humid for 1 year, Ojeda et al. (2009) found formation of biofilms comprising species of *Aspergillus* and *Penicillium*. Moreover, Moore-Kucera et al. (2014) reported growth of majority of members belonged to the family Trichocomaceae including species of *Aspergillus* and *Penicillium* on BP mulch surfaces in field soil buried for 6 months.

## Fungal mechanism for plastic degradation

Plastic biodegradation requires fungi to metabolize all organic components of the polymer which involves several key steps (Supplementary Fig. 4).

### Attachment on plastic surface by hydrophobin

According to previous studies, attachment of fungal cell on the polymer surface acts as the initial step in plastic decomposition process. The event is mainly mediated by hydrophobin, a family of low Mw (≤ 20 kDa), small and globular proteins secreted by filamentous fungi. They are characterized by eight cysteine residues organized in a conserved array with four disulphide bridges and typical amphipathic nature possessing both hydrophilic and hydrophobic parts (Berger and Sallada 2019). They are comprised of 70–350 amino acids including signal peptide sequence that helps in translocation of the protein across endoplasmic reticulum and eventually is cleaved by specific peptidases (Khalesi et al. 2015). The proteins are accumulated on the surface of mycelia, spores and aerial parts of fungi, as they play a crucial role in fungal growth and development as well as survival and adaptation in any environment (Linder et al. 2005). They are involved in formation of hydrophobic aerial structures and mediate attachment of hyphae to hydrophobic solid supports (Scholtmeijer et al. 2005). As a result, hydrophobins also help in anchoring mycelia on the polymer surface paving the way of biodegradation as illustrated by several researchers in case of LDPE deterioration by fungi (Santacruz-Juárez et al. 2021; Zahra et al. 2010) and such capacity makes the organism as better plastic degraders than bacteria (Ghatge et al. 2020). The attachment step eventually makes plastic film surface more hydrophilic which further aids in microbial colonization (Han et al. 2020).

### Colonization and biofilm formation

Biofilms are cellular consortia embedded in and/or attached to an extracellular polymeric matrix (Atanasova et al. 2021). It rapidly develops on any surface where water, nutrient and a source of carbon as well as energy are accessible. However, substratum properties such as hydrophobicity, crystallinity, roughness, melting temperature, elasticity modulus and glass transition temperature may influence selection of the microbial community during early stages of colonization. Previous studies have reported that agents like mineral oil and Tween 80 may improve hydrophobic interaction between the polymer and microbes, enhancing the rate of biofilm formation and degradation of PE (Devi et al. 2015). All in all, the prerequisite step for biodegradation is formation of biofilm on the plastic surface (De Tender et al. 2017).

After initial attachment of fungi to material surfaces, they can rapidly colonize and penetrate the substrates via their strong hyphal systems (Tamoor et al. 2021). Harding et al. (2009) indicated that filamentous fungi go through six steps for the development of biofilms: (a) deposition of spores on the surface of substrate (b) secretion of adhesive materials by spores and active germination (c) hyphal ramification (d) formation of dense hyphal and micelle network (e) enlargement in aerial growth of the colony, necessary for reproduction and (f) release of spores repeating the cycle.

### Primary plastic degradation through extracellular fungal enzymes

Fungi produce a wide range of extracellular and membrane bound enzymes that have the potency to break down chemical bonds of the plastic polymers (Supplementary Table 2). These proteins belong to two main classes, namely hydrolases and oxidases (Atanasova et al. 2021; Ren et al. 2021). Hydrolases (EC 3.1.x) are the group of enzymes that catalyze cleavage of chemical bond in presence of water resulting breakage of a larger molecule into smaller ones. Hydrophobic cleft present near active site of the enzyme can accommodate hydrophobic groups in the polymer facilitating accessibility of the enzyme to plastic (Kaushal et al. 2021). Two examples of hydrolases are esterases and lipases (EC 3.1.1.X) that can cleave ester bonds in the carbon chain and are active chiefly on aliphatic polyesters. Cutinases (EC 3.1.1.74) are a sub-class of esterase that possesses ability to hydrolyze polyesters with high molar mass and thus have gained increased attention. These carboxylic ester hydrolases were originally extracted from plant pathogen, *F. solani pisi* (Mohanan et al. 2020). Both these enzymes are attributed to degrade PET and PU owing to presence of hydrolysable chemical bond in the plastics (Temporiti et al. 2022). Recently, a 20.4 kDa BP-degrading esterase was isolated from *P. antarctica* JCM10317 that degraded PBSA and PCL more rapidly than PBS and PLA indicating preference of the enzyme for C6 acyl chains (Shinozaki et al. 2013a, b). Another BP-degrading cutinase similar esterase has also been isolated from *Paraphoma* sp. B47-9 that can degrade PBS, PBSA, and PCL films (Koitabashi et al. 2012, 2016). González-Márquez et al. (2021) reported that dibutyl phthalate, one of the most abundantly used plasticizers, can stimulate *Fusarium culmorum* and *Fusarium oxysporum* to release esterase in liquid culture indicating their anticipated utilization in the mitigation of environmental pollution. Proteases (EC 3.4.X) function to hydrolyze peptide and urethane bonds and thus are active on polyamides including different type of nylons (Atanasova et al. 2021). Another proteolytic enzyme is urease (EC 3.5.1.5) that has been enumerated to degrade polyester PU. Indeed, *Aspergillus*, *Trichoderma*, *Penicillium* and *Fusarium* sp. have been depicted to produce urease associated with PU degradation (Loredo-Treviño et al. 2011).

Peroxidases (EC 1.11.1.x) are a group of oxidoreductases that utilize H_2_O_2_ to catalyze oxidative reactions. Amongst the members, MnP (EC 1.11.1.13) and lignin peroxidase (LiP) (EC 1.11.1.14) play a significant role in biodegradation of complex polymeric constituents such as lignin that has structure similarity with synthetic plastics (Khatoon et al. 2017). MnP is a glycosylated heme containing enzyme that catalyzes oxidation of Mn^2+^ to Mn^3+^ in a H_2_O_2_-dependent reaction attacking both phenolic and non-phenolic compounds (Zhao et al. 2015). LiP is a monomeric hemoprotein with molecular mass of around 40 kDa that catalyzes H_2_O_2_-dependent oxidative depolymerization of lignin (Ghosh and Pal 2021). The protein activity was first observed in case of *Phanerochaete chrysosporium* growing under limited nitrogen condition. LiP in general operates at an optimal reaction condition of 25–35 °C and 2–5 pH (Khatoon et al. 2017). Another pertinent fungal enzyme is laccase (EC 1.10.3.2), a blue and multicopper containing protein, that catalyzes one-electron oxidation of a broad range of phenolic compounds as well as aromatic amines by reducing molecular oxygen to water (Zeghal et al. 2021). Fujisawa et al. (2001) have reported that lac-mediator system along with 1-hydroxybenzotriazole as a mediator could degrade high Mw PE and nylon-66 membrane. Several studies have demonstrated the production of majority of laccase from various higher fungi like *Coriolopsis polyzona*, *Cerrena maxima*, *Lentinus tigrinus*, *Pleurotus eryngii*, *Trametes* sp., and among others. Additionally, laccases occur in some saprophytic Ascomycetes as well, such as *Myceliophthora thermophila* and *Chaetomium thermophile* (Sumathi et al. 2016). Both laccase and peroxidases input O_2_ in the main chain of PE and disturb strong electrical balance within polymer structure. As a result, carbonyl groups are formed and CH_2_ chain becomes hydrophilic making it liable to enzymatic break down (Ghosh and Pal 2021). Recent studies have shown that both Ascomycota and Basidiomycota can produce oxygenase where a large number of white-rot fungi, including *Phanerochaete chrysosporium*, *Trametes versicolor*, *Pleurotus ostreatus*, *Bjerkandera* sp. and others, have been reported to produce both MnP and LiP (Kang et al. 2019; Khatoon et al. 2019; Zhao et al. 2015). While, *Phlebia radiata* has been ascribed to produce Laccase and LiP (Kantelinen et al. 1989). It has been reported that PE can be effectively degraded through the use of enzymes such as amylase, laccase, LiP and MnP. These enzymes can cleave their carbon bonds, create functional groups and enable utilization of the PE films by the microorganisms. Nevertheless, fungi can produce other enzymes also such as glucosidase, cellulase, pectinase and hemicellulase that may play a remarkable role in degradation of polymers in soil resulting formation of small fragments (El-Morsy et al. 2017). All these enzymes are able to cleave polymeric materials into water soluble, small fragmented elements of 10–50 carbon atoms with concomitant production of highly reactive free radicals. These short chains are then transported into the cell for further metabolism (Khatoon et al. 2019). Overall, enzymatic degradation is an optimal plastic trash treatment strategy, particularly from industrial point of view, which not only hastens the process in a controllable mean but also recycles hydrolysate (Li et al. 2011).

### Final degradation of plastic

It is considered that five functional groups namely hydroxyl, carbonyl, carboxyl, sulfhydryl and phosphate are essential to support the chemistry of life. Micro(nano)plastics having any of them may thus easily enter into the fungal cells owing to their small size (Maity et al. 2021). Assimilation of higher *n*-alkanes is common amongst Ascomycetous and Basidiomycetous species and this is mainly believed to be due to the action of cytochromes P450, especially CYP52 family members (Cowan et al. 2022). CYP450 monooxygenases hydroxylate both alkanes and polyaromatic hydrocarbons, but their oxidized derivatives are then degraded via different mechanisms. The oxidized alkanes undergo β-oxidation and are further oxidized resulting in formation of the final products like CO_2_ and biomass (Ganesh Kumar et al. 2020). Indeed, synthetic polymers act as potent energy and carbon sources for microbial organisms. It has been deduced that complete oxidation of PE produces usable energy varying from − 422 to − 425 kJ per mole of O_2_. On the other hand, the usable energy generated from glucose is − 479 kJ per mole O_2_ indicating complete oxidation of PE results around the same energy as glucose (Asiandu et al. 2021).

As a consequence, the physicochemical properties of plastic films modify; evident by alterations in functional groups, crystallinity and Mw distribution. However, the magnitude of degradation is widely assessed by calculating weight loss of the plastic polymer. Special emphasis is also given to the carbonyl index (CI), i.e., ratio of absorbance peak at 1712 cm^–1^ to that of CH_2_ at 1462 cm^–1^ (Maity et al. 2021). On the other hand, decrease of water contact angle (WCA) is a hallmark of hydrophilicity increment of the polymer surface that can be detected by a contact angle measuring device (Awasthi et al. 2017). Besides, plastic superficial topography can be observed using SEM to identify surface destruction represented by formation of pits, scars, cracks, holes, erosions and cavities (Han et al. 2020). It also results in increase of TDS of media which is the sum of all organic and inorganic substances such as salts and various nutrients present in colloidal, molecular or suspended form (Chaudhary and Vijayakumar 2020a). Consequently, consumption of O_2_ (respirometric test) or estimation of the end product, CO_2_, (Sturm test) are good indicators for plastic deterioration and thus are the frequently used laboratory tests to designate biodegradation (Osman et al. 2018).

## Conclusion and directions for future research

Plastics are versatile materials offering many benefits for the future; despite that, the present solutions addressing plastic waste crisis are neither adequate nor eco-friendly. To solve the problem related to plastic waste accumulation, production of BPs from suitable renewable resources is a fruitful notion. Use of these substances should be recommended especially for food item packaging and manufacturing agricultural films, fishery materials as well as medical items. However, suitable management of their leftover and littering control is also crucial to take benefit of such polymers in the community. Thus, the search for competent, sustainable and low-cost waste management technology is in continuation.

In this context, biodegradation of plastics by fungal strains could be the most innovative and economically as well as environmentally beneficial way to tackle the waste problem and reduce native impact on the environment. Till date, many Ascomycetous fungal strains namely *Aspergillus*, *Candida*, *Fusarium*, *Cladosporium*, *Paecilomyces*, *Penicillium* and so forth have been acknowledged as strong degraders of a widespread range of plastics where most of the studies have been published focusing mainly on degradation of PE by fungi; while fate of other polymer types as well as micro-nano forms remain comparatively neglected. Besides, lesser attention has been paid on elucidation of the mechanism of action which so far been understood to occur through attachment of fungal hyphae on polymer surface by hydrophobin, formation of biofilm and secretion of extensive enzymes that can break down polymers into small fragments. Indeed, research suggests that PE including HDPE and LDPE as well as PVC, PS and nylon can be decomposed by laccase; while PBS, PBSA, PCL and PET can be deteriorated by ester hydrolases, i.e., lipases, esterases and cutinases synthesized by fungi. Enzyme engineering thus may aid to speed up biodegradation process as long period of the method is a key limitation for practical application. Some researchers also have illustrated that overexpression of these enzymes in heterologous host and genetic modifications can be helpful to improve natural capacity of fungi for plastic degradation; however, real-world application using high population of such mutant types may cause an imbalance in the ecosystem.

The present review also highlights that the total number of investigated specimens is much lower than the actual number of fungi present around the globe indicating many taxa still remain unexplored. It might be due to the prevalence of uncultured fungal species creating challenge to identify them from the respective plastic degrading environment. To overcome this problem, metagenomic study of microbial population in plastishere is highly advisable as in nature they might be working synergistically rather than individually. Further, bio-augmentation of potant organisms is necessary that can be spread over the plastic contaminated place to mitigate the problem. For better consideration, high-throughput omics approaches such as meta-genomics, meta-transcriptomics, meta-proteomics, metabolomics, fluxomics and bioinformatics investigations can be employed. These methods may open up the scope for mining genes or enzymes engaged in polymer degradation. Thus, further research on myco-diversity, enzymatic degradation mechanisms and related metabolism will be necessary to reduce global plastic pollutants and offer good health for future generations.

### Supplementary Information

Below is the link to the electronic supplementary material.

Supplementary material 1 (DOC 3965.0 kb)
