# Supplementary material for: Myco-remediation of plastic pollution: current knowledge and future prospects
Source: Biodegradation. 2023 Sep 4;35(3):249–79. doi: 10.1007/s10532-023-10053-2 (PMC10950981; doi:10.1007/s10532-023-10053-2)
Supplement: Supplementary file 1 — Supplementary material 1 (DOC 3965.0 kb) [file 10532_2023_10053_MOESM1_ESM.doc]

Supplementary material:

**Myco-remediation of plastic pollution: Current knowledge and future prospects**

Somanjana Khatua^a^, Jesus Simal-Gandara^b^*, Krishnendu Acharya^c^**

^a^Department of Botany, Faculty of Science, University of Allahabad, Prayagraj 211002, Uttar Pradesh, India

^b^Universidade de Vigo, Nutrition and Bromatology Group, Department of Analytical Chemistry and Food Science, Faculty of Science, E-32004, Ourense, Spain

^c^Molecular and Applied Mycology and Plant Pathology Laboratory, Centre of Advanced Study, Department of Botany, University of Calcutta, 35, Ballygunge Circular Road, Kolkata, 700019, West Bengal, India

*Corresponding author e-mail: jsimal@uvigo.es (J. Simal-Gandara)

**Corresponding author e-mail: [krish_paper@yahoo.com](mailto:krish_paper@yahoo.com) (K. Acharya)


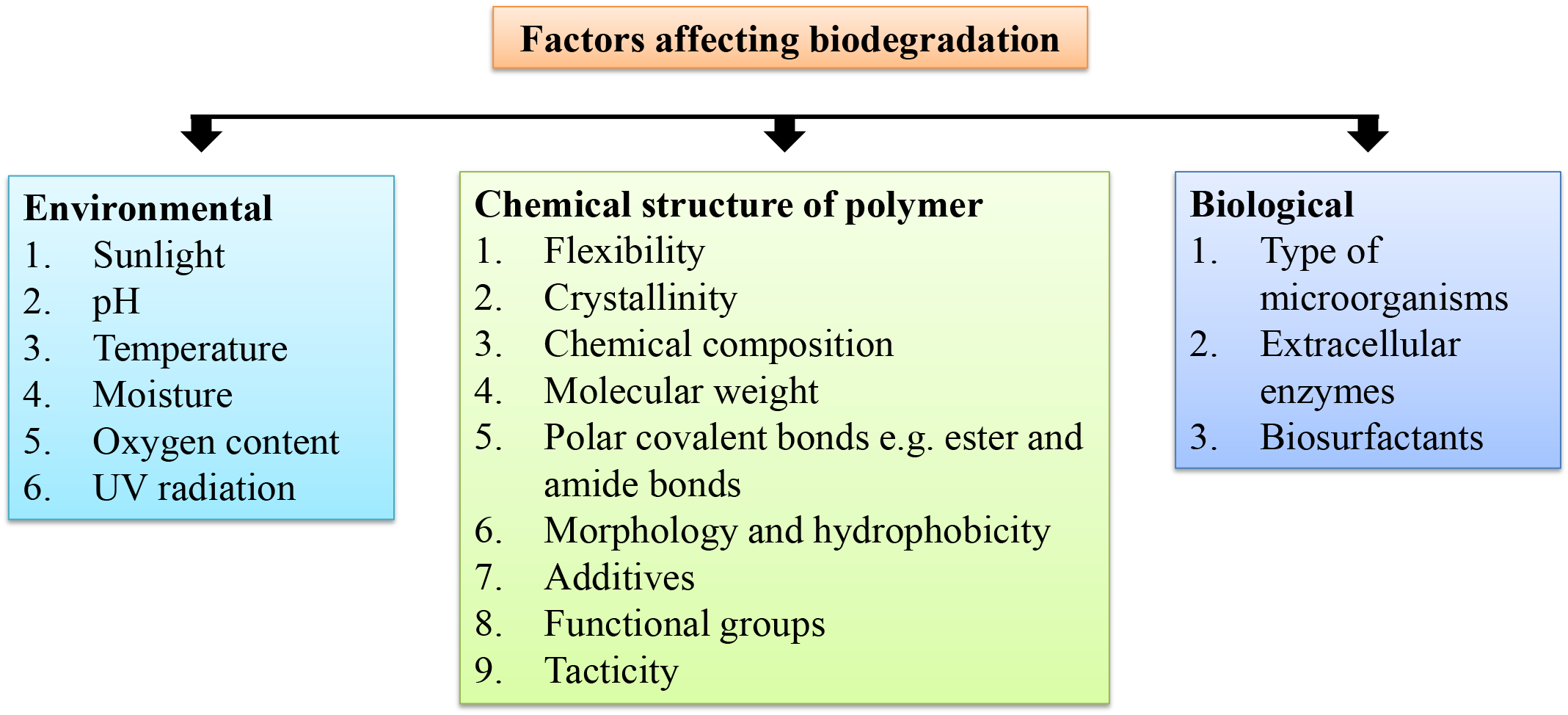


**Supplementary Figure 1:** Factors affecting rate of plastic biodegradation.

**Supplementary Table 1:** GenBank accession of specimens included in molecular phylogenetic analyses of plastic degrading fungi.

| Sl no | Taxon | Plastic type | ITS accession number | Size (bp) |
| --- | --- | --- | --- | --- |
| 1 | *Actinomucor elegans* | Biodegradable plastic | NR_173404.1 | 686 |
| 2 | *Agaricus bisporus* | PU | AJ409229.1 | 671 |
| 3 | *Alternaria alternata* | LDPE | AJ853759.1 | 528 |
| 4 | *Alternaria tenuissima* | PU | AJ867284.1 | 551 |
| 5 | *Aspergillus caespitosus* | LDPE | AY373841.1 | 594 |
| 6 | *Aspergillus clavatus* | LDPE | NR_121482.1 | 710 |
| 7 | *Aspergillus flavus* | HDPE, LDPE, PS | KX463034.1 | 560 |
| 8 | *Aspergillus fumigatus* | LDPE, PP, PU, biodegradable plastic | NR_121481.1 | 707 |
| 9 | *Aspergillus oryzae* | LDPE | NR_135395.1 | 616 |
| 10 | *Aspergillus nidulans* | LDPE | NR_133684.1 | 547 |
| 11 | *Aspergillus niger* | LDPE, HDPE, PVC, PU, PS | FJ878652.1 | 623 |
| 12 | *Aspergillus nomius* | LDPE | DQ370001.1 | 574 |
| 13 | *Aspergillus sydowii* | PE, PVC | NR_131259.1 | 510 |
| 14 | *Aspergillus terreus* | PE, LDPE, HDPE | KM116156.1 | 479 |
| 15 | *Aspergillus tubingensis* | HDPE, PU | HQ905466.1 | 550 |
| 16 | *Aspergillus versicolor* | LDPE | NR_131277.1 | 591 |
| 17 | *Aureobasidium pullulans* | PVC, PU, biodegradable plastic | NR_144909.1 | 486 |
| 18 | *Bjerkandera adusta* | PP, nylon, biodegradable plastic | MW182414.1 | 613 |
| 19 | *Candida tropicalis* | LDPE, Biodegradable plastic | NR_111250.1 | 493 |
| 20 | *Chaetomium globosum* | PVC | NR_144851.1 | 529 |
| 21 | *Cladosporium cladosporioides* | PU | NR_119839.1 | 640 |
| 22 | *Cladosporium herbarum* | PU | NR_119656.1 | 509 |
| 23 | *Cladosporium subcinereum* | Biodegradable plastic | NR_148193.1 | 543 |
| 24 | *Clonostachys rosea* | Biodegradable plastic | NR_165993.1 | 568 |
| 25 | *Curvularia senegalensis* | PU | MT410577.1 | 560 |
| 26 | *Cymatoderma dendriticum* | PS | OL771705.1 | 686 |
| 27 | *Diaporthe italiana* | LDPE | NR_165892.1 | 566 |
| 28 | *Fusarium falciforme* | PE | NR_164424.1 | 586 |
| 29 | *Fusarium solani* | LDPE, PU | NR_163531.1 | 632 |
| 30 | *Geomyces pannorum* | PU | KF153032.1 | 499 |
| 31 | *Lasiodiplodia theobromae* | LDPE, PP | NR_111174.1 | 542 |
| 32 | *Lentinus tigrinus* | PVC | KY565250.1 | 637 |
| 33 | *Paecilomyces lilacinus* | LDPE | AB558286.1 | 495 |
| 34 | *Paecilomyces variotii* | LDPE | EU037050.1 | 568 |
| 35 | *Penicillium chrysogenum* | LDPE, HDPE | NR_077145.1 | 585 |
| 36 | *Penicillium griseofulvum* | PU | NR_103692.1 | 584 |
| 37 | *Penicillium janthinellum* | PVC | NR_111504.1 | 548 |
| 38 | *Penicillium oxalicum* | LDPE, HDPE | NR_121232.1 | 611 |
| 39 | *Penicillium pinophilum* | LDPE | JQ003471.1 | 542 |
| 40 | *Penicillium simplicissimum* | PE, LDPE | NR_138290.1 | 547 |
| 41 | *Pestalotiopsis microspora* | PU | AY681483.1 | 590 |
| 42 | *Phanerochaete chrysosporium* | LDPE, PVC | MH854905.1 | 639 |
| 43 | *Phialophora alba* | LDPE | HM116755.1 | 624 |
| 44 | *Pleurotus ostreatus* | Biodegradable plastic | NR_163515.1 | 630 |
| 45 | *Pleurotus sajor caju* | PVC | DQ077890.1 | 639 |
| 46 | *Purpureocillium lilacinum* | PE | NR_165946.1 | 578 |
| 47 | *Rhizopus arrhizus* | LDPE | NR_103595.1 | 610 |
| 48 | *Stagonosporopsis citrulli* | LDPE | MT040723.1 | 498 |
| 49 | *Talaromyces funiculosus* | PET | NR_103678.2 | 612 |
| 50 | *Thermomyces lanuginosus* | LDPE | NR_121309.1 | 634 |
| 51 | *Trametes versicolor* | PVC | NR_154494.1 | 701 |
| 52 | *Trichoderma viride* | LDPE | AY380907.1 | 517 |
| 53 | *Yarrowia lipolytica* | PP | NR_111212.1 | 317 |
| 54 | *Zalerion maritimum* | PE | AF169305.1 | 556 |


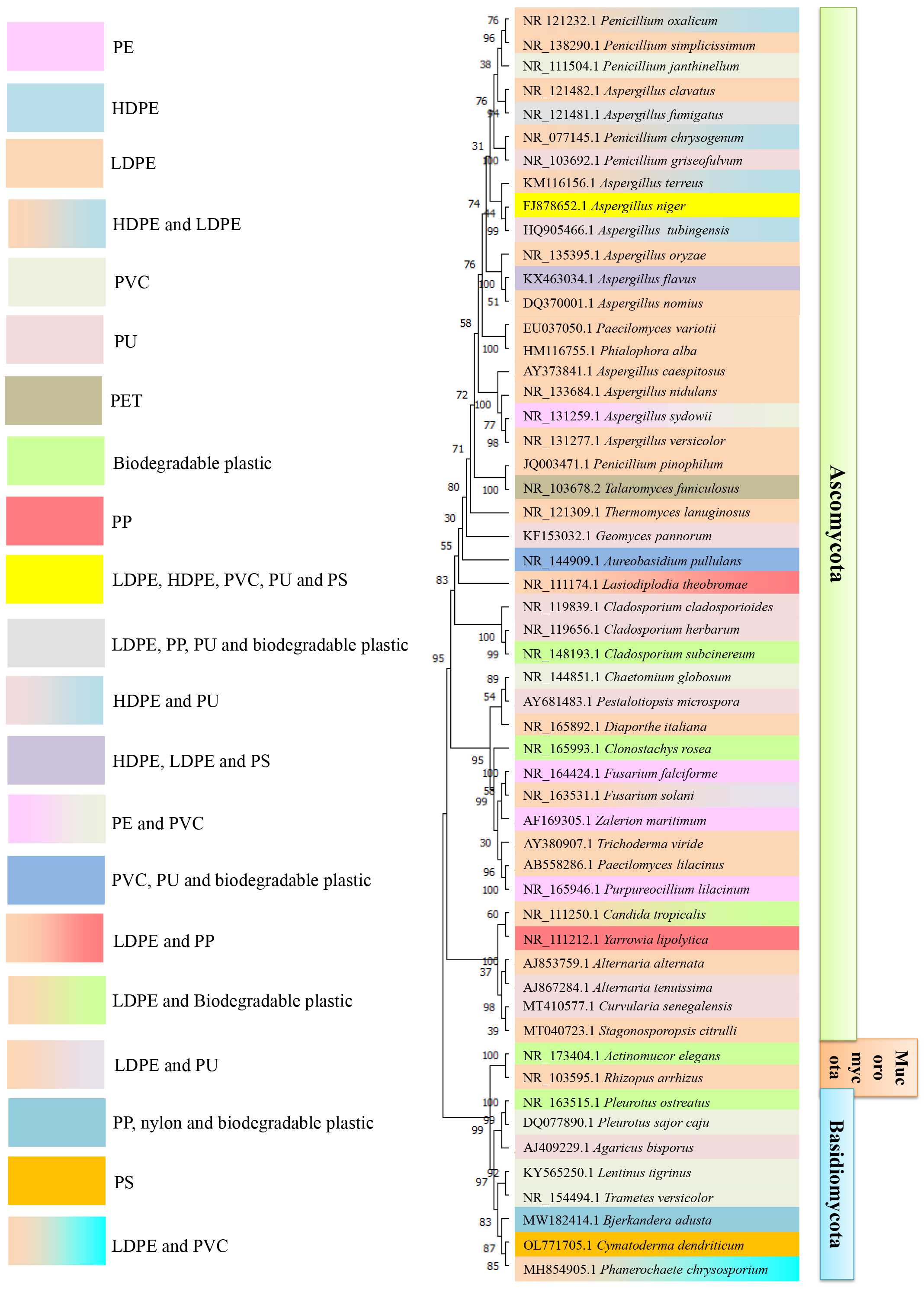


**Supplementary Figure 2:** Phylogenetic positions of plastic degrading fungi based on comparison of nucleotide sequences of the 28S rRNA gene were retrieved using Maximum-Likelihood method and Kimura 2-parameter model with the help of Mega X. Numbers at the nodes indicate bootstrap values from the analysis of 1000 resampled data sets.


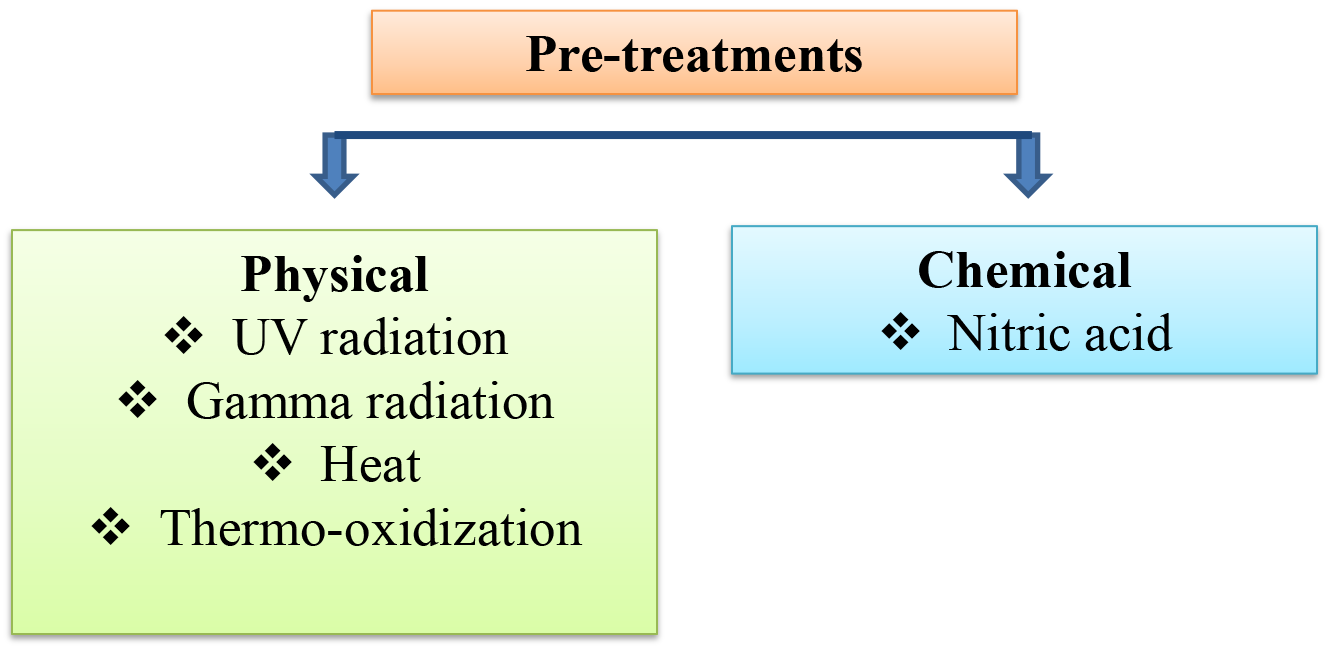


**Supplementary Figure 3:** Pre-treatment methods implemented in some researches before biodegradation by fungi (Chaudhary and Vijayakumar, 2020a; Mathur *et al*. 2011).

**Supplementary Table 2.** Fungal enzymes involved in biodegradation of plastics of different kinds.

| **Fungi** | **Enzyme** | **Plastic type** | **Reference** |
| --- | --- | --- | --- |
| *Alternaria alternata* FB1 | Laccase (59.49 kDa), peroxidase (36.7 kDa) | PE | Gao et al. 2022 |
| *Alternaria alternata*, *Aspergillus* *caespitosus*, *Aspergillus terreus*, *Paecilomyces variotii*, *Phialophora alba* | Laccase, MnP, LiP | LDPE | Ameen et al. 2015 |
| *Agaricus bisporus, Pleurotus abalones*, *Pleurotus ostreatus* | Laccase | PE, PS | Hock et al. 2019 |
| *Aspergillus flavus* | Laccase like multicopper oxidase | HDPE MPs | Zhang et al. 2020 |
| *Aspergillus flavus* | Glycosidase | PCL | Tokiwa et al. 2009 |
| *Aspergillus oryzae* | Cutinase | PBS and PBSA films | Maeda et al. 2005 |
| *Aspergillus niger* | Catalase, protease | PCL | Tokiwa et al. 2009 |
| *Aspergillus niger, Diaporthe italiana, Stagonosporopsis citrulli, Thyrostroma jaczewskii* | Laccase, MnP, LiP | LDPE | Khruengsai et al. 2021 |
| *Aspergillus oryzae* RIB40 | Cutinase | PDLLA | Maeda et al. 2005 |
| *Aspergillus tubingensis* | Lipase, esterase | PU | Khan et al. 2017 |
| *Aspergillus* sp.*, Lasiodiplodia theobromae*, *Paecilomyces lilacinus* | Laccase | LDPE | Sheik et al. 2015 |
| *Aspergillus* sp*.* | Esterase | PU | Osman et al. 2017 |
| *Aspergillus* sp. XH0501-a | Unknown (44.7 kDa) | PBS | Li et al. 2011 |
| *Aureobasidium pullulans* | Esterase | PVC | Webb et al. 2000 |
| *Bjerkandera adusta* | MnP | Nylon-6 fiber | Friedrich et al. 2007 |
| *Candida antarctica* | Lipase | PCL | Shi et al. 2020 |
| *Cochliobolus* sp. | Laccase | PVC | Sumathi et al. 2016 |
| *Cryptococcus* sp. strain S-2 | Cutinase | PDLA | Masaki et al. 2005 |
| *Curvularia senegalensis* | Esterase (28 kDa) | PU | Crabbe et al. 1994 |
| *Eupenicillium hirayamae* | Laccase, MnP | LDPE | Ameen et al. 2015 |
| *Fusarium solani* | Esterase, lipase | PU | Ren et al. 2021 |
| *Fusarium solani* | Cutinase | PET | Eberl et al. 2009 |
| *Fusarium solani* | Cutinase | PCL | Shi et al. 2020; Murphy et al. 1996 |
| *Fusarium solani, Humicola insolens* | Cutinase | PET | Ronkvist et al. 2009 |
| *Microsphaeropsis arundinis* | Lipase, esterase | PET | Malafatti-Picca et al. 2019 |
| *Monascus ruber*, *Monascus sanguineus*, *Monascus* sp. | Esterase | PU | El-Morsy et al. 2017 |
| *Paraphoma* like fungus strain B47-9 | PCLE (19.7 kDa) | PBSA | Suzuki et al. 2014 |
| *Paraphoma* like fungus | Unknown | BPs | Sameshima-Yamashita et al. 2016 |
| *Paraphoma* sp. B47-9 | BP-degrading enzyme (19.9 kDa) | PBS, PBSA, PCL films | Koitabashi et al. 2016; Koitabashi et al. 2012 |
| *Penicillium citrinum* | Polyesterase (14.1 kDa) | PET | Liebminger et al. 2007 |
| *Penicillium funiculosum* | Unknown | PHB | Tokiwa et al. 2009 |
| *Penicillium simplicissimum* | Lipase | LDPE | Ghosh and Pal, 2021 |
| *Penicillium simplicissimum* | Laccase (66 kDa), MnP (60 kDa) | Pre-treated LDPE | Sowmya et al 2015 |
| *Pestalotiopsis microspora* | Serine hydrolase (~21 kDa) | PU | Russell et al. 2011 |
| *Penicillium oxalicum* SS2 | PHBV depolymerase (36 kDa) | PHB, PHBV | Satti et al. 2020 |
| *Phanerocheate chrysosporium* | LiP (46 kDa) | PVC | Khatoon et al. 2019 |
| *Phanerochaete chrysosporium* | MnP | PE | Shimao 2001 |
| *Pleurotus ostreatus* | Laccase | Oxo-BPs | da luz et al. 2013 |
| *Pseudozyma antarctica* | Lipase B | PCL | Shinozaki et al. 2013b |
| *Pseudozyma antarctica*  JCM 10317 | Unknown (~22 kDa) | BPs | Kitamoto et al. 2011 |
|  | Esterase (20.4 kDa) | PBS and PBSA |  |
|  |  |  |  |
| *Paecilomyces lilacinus* | PHB depolymerase | PHB | Oda et al. 1995 |
|  | PCL depolymerase | PCL |  |
| *Rhizopus delemer* | Lipase | Copoly(d-lactic acid/l-lactic acid | Fukuzaki et al. 1989 |
| *Trametes versicolor* | Laccase | Nylon, PE | Fujisawa et al. 2001 |
| *Trichoderma harzianum* | Laccase (88 kDa), MiP (55 kDa) | PE | Sowmya et al. 2014 |
| *Trichoderma viride* | Laccase | LDPE | Johnnie et al. 2021 |
| *Trichoderma* sp*.* | Urease | PU | Loredo-Treviño et al. 2011 |
| *Tritirachium album* ATCC 22563 | Protease | PLLA | Jarerat and Tokiwa, 2001 |
| *Yarrowia lipolytica* IMUFRJ 50682 | Lipase | PET | Da Costa et al. 2020 |

PHBV: poly(3-hydroxybutyrate-co-3-hydroxyvalerate)


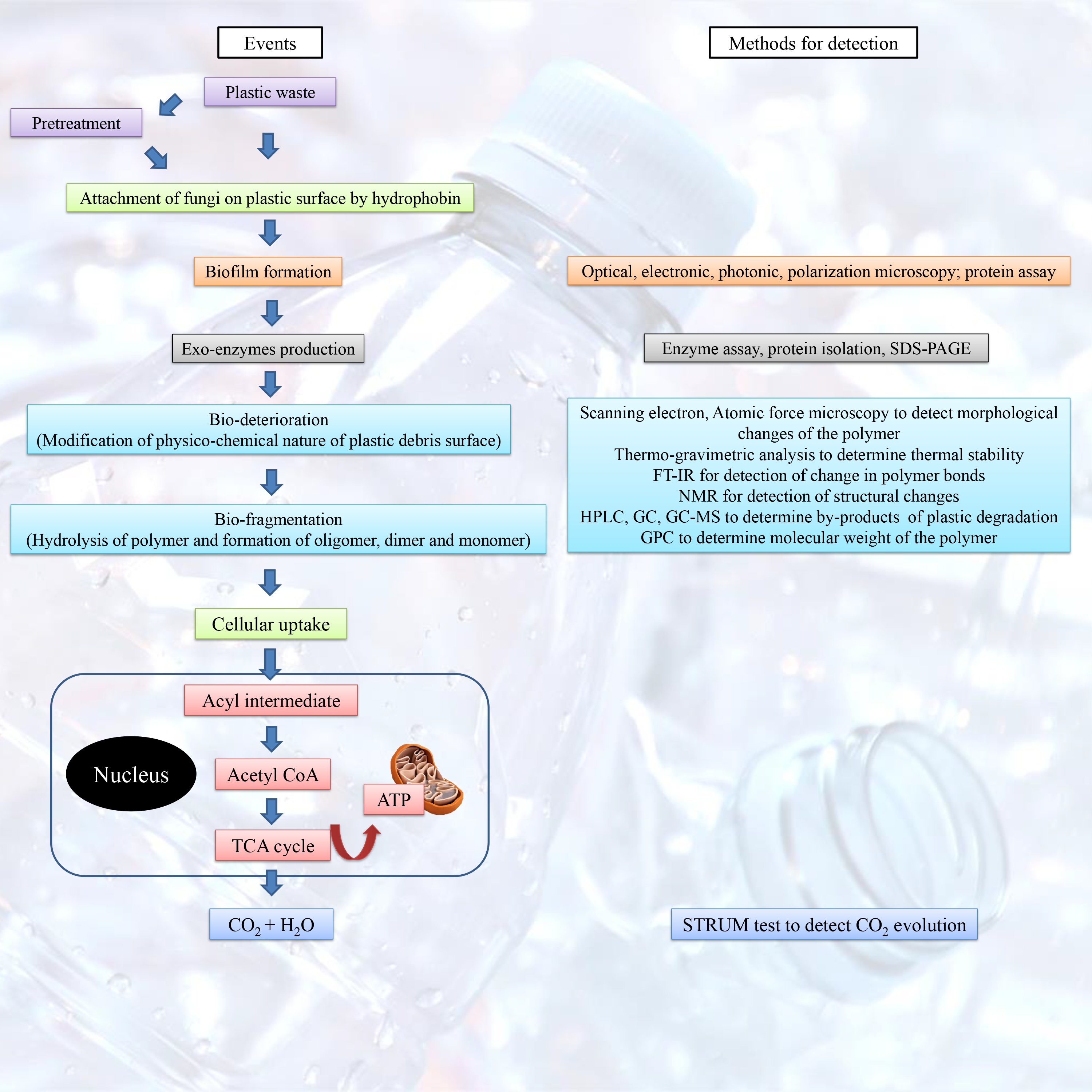


**Supplementary Figure 4:** Schematic representation of mechanism of action (left side) associated with the process of plastic degradation by fungi. An overview of laboratory test methods (right side) has also been incorporated that are widely performed to understand potentiality of studied organism to decompose plastics.
